# Mutations in *Kinesin family member 6* reveal specific role in ependymal cell ciliogenesis and human neurological development

**DOI:** 10.1371/journal.pgen.1007817

**Published:** 2018-11-26

**Authors:** Mia J. Konjikusic, Patra Yeetong, Curtis W. Boswell, Chanjae Lee, Elle C. Roberson, Rungnapa Ittiwut, Kanya Suphapeetiporn, Brian Ciruna, Christina A. Gurnett, John B. Wallingford, Vorasuk Shotelersuk, Ryan S. Gray

**Affiliations:** 1 Department of Pediatrics, Dell Pediatric Research Institute, The University of Texas at Austin, Dell Medical School, Austin, Texas, United States of America; 2 Department of Molecular Biosciences, Patterson Labs, The University of Texas at Austin, Austin, Texas, United States of America; 3 Center of Excellence for Medical Genetics, Department of Pediatrics, Faculty of Medicine, Chulalongkorn University, Bangkok, Thailand; 4 Excellence Center for Medical Genetics, King Chulalongkorn Memorial Hospital, the Thai Red Cross Society, Bangkok, Thailand; 5 Division of Human Genetics, Department of Botany, Faculty of Science, Chulalongkorn University, Bangkok, Thailand; 6 Program in Developmental & Stem Cell Biology, The Hospital for Sick Children, Toronto, Ontario, Canada; Department of Molecular Genetics, The University of Toronto, Toronto, Ontario, Canada; 7 Department of Neurology, Division Pediatric Neurology, Washington University School of Medicine, St Louis, MO, United States of America; University of Massachusetts Medical School, UNITED STATES

## Abstract

Cerebrospinal fluid flow is crucial for neurodevelopment and homeostasis of the ventricular system of the brain, with localized flow being established by the polarized beating of the ependymal cell (EC) cilia. Here, we report a homozygous one base-pair deletion, c.1193delT (p.Leu398Glnfs*2), in the *Kinesin Family Member 6* (*KIF6*) gene in a child displaying neurodevelopmental defects and intellectual disability. To test the pathogenicity of this novel human *KIF6* mutation we engineered an analogous C-terminal truncating mutation in mouse. These mutant mice display severe, postnatal-onset hydrocephalus. We generated a *Kif6-LacZ* transgenic mouse strain and report expression specifically and uniquely within the ependymal cells (ECs) of the brain, without labeling other multiciliated mouse tissues. Analysis of *Kif6* mutant mice with scanning electron microscopy (SEM) and immunofluorescence (IF) revealed specific defects in the formation of EC cilia, without obvious effect of cilia of other multiciliated tissues. Dilation of the ventricular system and defects in the formation of EC cilia were also observed in adult *kif6* mutant zebrafish. Finally, we report Kif6-GFP localization at the axoneme and basal bodies of multi-ciliated cells (MCCs) of the mucociliary *Xenopus* epidermis. Overall, this work describes the first clinically-defined *KIF6* homozygous null mutation in human and defines KIF6 as a conserved mediator of neurological development with a specific role for EC ciliogenesis in vertebrates.

## Author summary

Cerebrospinal fluid flow is crucial for neurodevelopment and homeostasis of the ventricular system of the brain. Localized flows of cerebrospinal fluid throughout the ventricular system of the brain are established from the polarized beating of the ependymal cell (EC) cilia. Here, we identified a homozygous truncating mutation in *KIF6* in a child displaying neurodevelopmental defects and intellectual disability. To test the function of KIF6 *in vivo*, we engineered mutations of *Kif6* in mouse. These *Kif6* mutant mice display severe hydrocephalus, coupled with defects in the formation of EC cilia. Similarly, we observed hydrocephalus and a reduction in EC cilia in *kif6* mutant zebrafish. Overall, this work describes the first clinically-defined *KIF6* mutation in human, while our animal studies demonstrate the pathogenicity of mutations in *KIF6* and establish KIF6 as a conserved mediator of ciliogenesis in ECs in vertebrates.

## Introduction

The delicate balance of cerebrospinal fluid (CSF) production and flow is important for the morphogenesis and function of the brain during development and homeostasis. CSF circulation in human is largely due to gradients established by the secretion of CSF from the choroid plexuses, and its resorption at the arachnoid granulations [1]. The clinical significance of CSF stasis includes hydrocephalus and intracranial hypertension. Moreover, severely diminished CSF flow combined with increased intracranial pressure can secondarily cause ventriculomegaly, cognitive impairment, as well as degenerative and age-related dementias [2]. For these reasons, the identification of genetic risk factors involved in the pathogenesis of CSF stasis is critical for the development of genetic diagnostics and early interventions for these disorders.

One element for circulation of CSF is the multiciliated ependymal cells (ECs), which are specialized glial cells covering the ventricular walls of the brain and spinal canal [3]. In contrast, to primary cilia which are single, immotile cellular organelles extending from most cell types, ECs contain dozens of apically-arranged motile cilia, which beat in a polarized fashion to generate localized or near-wall CSF flows [4]. Defective differentiation or alterations in their stereotyped synchronous, polarized beating leads to alterations of localized CSF flow contributing to increased intracranial pressure, dilation of ventricles, and hydrocephalus in mice [5–8]. Importantly, this EC cilia-driven CSF flow is vital for regulating brain function and neurogenesis during adult development [4, 9].

Impaired motility of cilia due to disruptions of the key kinesins, dyenins, or intraflagellar components, are associated with a syndromic condition known as primary ciliary dyskinesia (PCD) in humans [10, 11]. While hydrocephalus can occur in some PCD patients, it is a less common manifestation of the disease in humans [11]. In contrast, genes implicated in PCD or mutations which disrupt the structure or motility of all motile cilia are strongly correlated with hydrocephalus in mouse [8]. Alternatively, some hydrocephalus in mice with dysfunctional cilia may be the result of altered function of the choroid plexus, prior to the onset of cilia-driven CSF flow [7].

*KIF6* (Kinesin family member 6, OMIM: 613919) encodes a member of the kinesin-9 superfamily of microtubules motor proteins which act predominately as "plus-end" directed molecular motors that generate force and movement across microtubules [12]. Kinesins are critical for numerous cellular functions such as intracellular transport and cell division, as well as for building and maintaining the cilium in a process known as intraflagellar transport [13]. During this process, kinesins have been shown to transport cargo within the ciliary axoneme [14], establish motility and compartmentalization of the axoneme [15], or to facilitate plus-end directed microtubule disassembly and control of axonemal length [16]. As such, multiple kinesins have shown to be associated with monogenic disorders affecting a wide-spectrum of tissues, with several modes of inheritance (www.omim.org). Interestingly, *KIF6* has previously been proposed as locus for susceptibility to coronary heart disease [17], while other studies did not substantiate this association [18]. We previously reported that *kif6* mutant zebrafish are adult viable exhibiting larval-onset scoliosis without obvious heart defects [19]. Because of these conflicting results, and a lack of relevant mouse models, the role of KIF6 in human disease remains an open question.

Here, we present a patient with consanguineous parents, presenting with abnormal neurological morphologies and intellectual disability. Homozygosity mapping followed by whole-exome sequencing (WES) identified a novel homozygous frameshift mutation in *KIF6* which is predicted to result in the truncation of the C-terminal cargo-binding domain of the kinesin motor protein. We generated an analogous frameshift mutation in mouse and found that these mutant mice displayed progressive, postnatal-onset hydrocephalus with cranial expansion, coupled with an obvious defect in EC cilia formation. In addition, we observed that *kif6* mutant zebrafish also display dilation of the ventricular system, coupled with reduced EC cilia. We failed to observe cilia defects in other multiciliated tissues in *Kif6* mutant mouse or zebrafish models. Together these results demonstrate that KIF6 has a specific function for the formation of EC cilia. Finally, we propose that *KIF6* represents a novel locus for understanding mechanisms of neurological development and intellectual disability in humans.

## Results

### Clinical features and mutation identification

**Fig 1:**
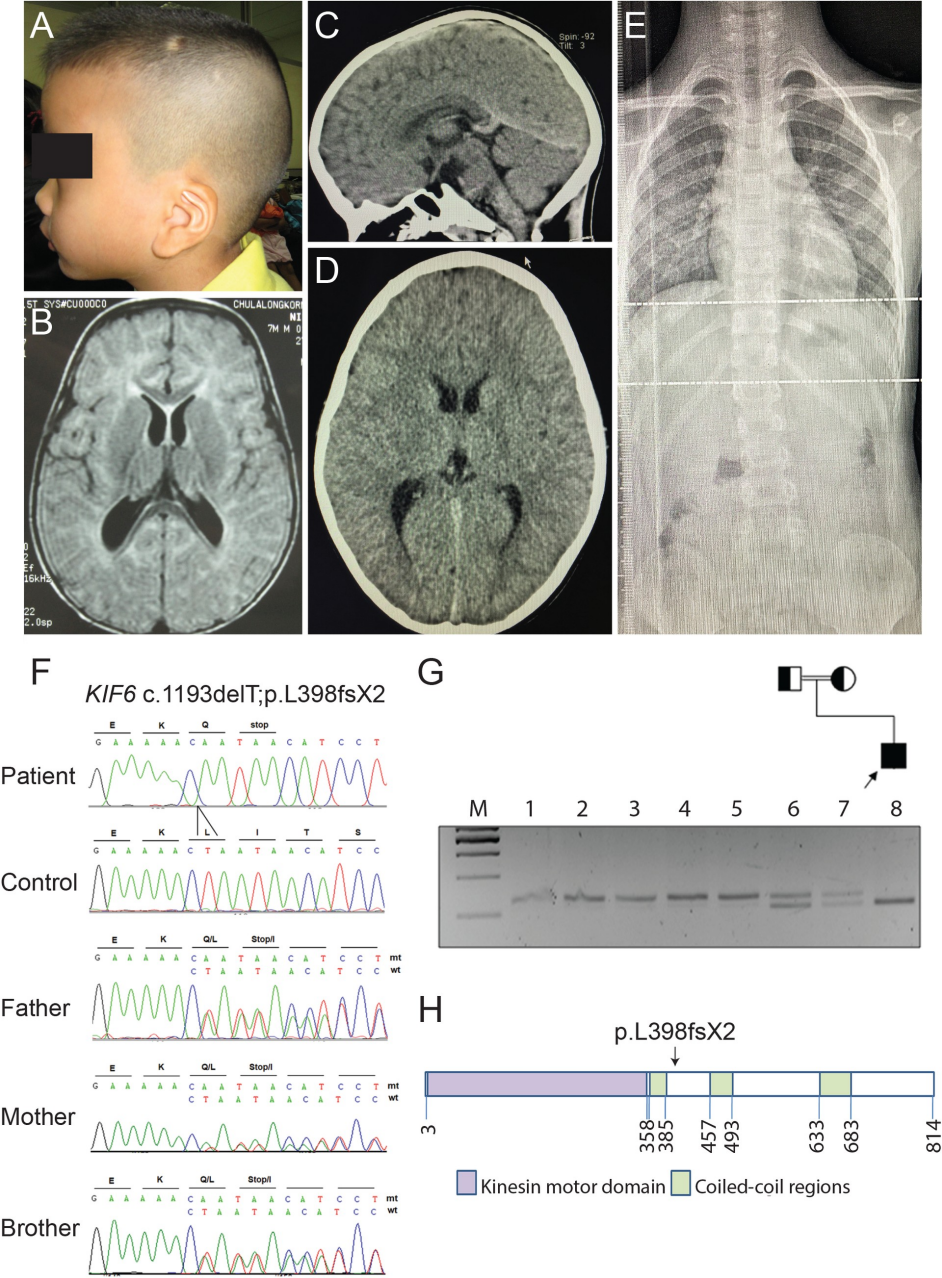
*KIF6* mutation in a child with intellectual disability. (A) A low-set prominent anti-helical left pinna. (B) MRI of the brain at 7 months-old shows dolichocephaly with a normal brain structure. (C) and (D) CT of the brain at 8 years-old, sagittal and axial views, respectively show dolichocephalic shape of the cranium (cephalic index = 75) without demonstrable intracranial abnormality. (E) X-ray of the spine shows no scoliosis (F) Electropherograms of the patient, a control, the patient’s father, mother, and unaffected brother from top to bottom. The patient is homozygous for the c.1193delT mutantion while his father, mother, and unaffected brother are all heterozygous carriers. (G) Pedigree and RFLP, using MfeI restriction enzyme: Lane M = 100 bp marker. The arrow head indicates the 500 bp band. Lanes 1–5 are controls. Lanes 6 and 7 are the proband’s father and mother, respectively, showing that they are heterozygous. Lane 8 is the proband showing that he is homozygous for the c.1193delT. (H) Representative KIF6 structure. The arrow shows the position of the c.1193delT mutation.

We identified a Thai boy with intellectual disability and megalencephaly. His parents were first cousins. He was born at 34 weeks gestation with a head circumference of 34 cm (97^th^ centile). APGAR scores were 7 and 9 at 1 and 5 minutes, respectively. Neonatal hypoglycemia (blood sugar of 11 mg/dL) and neonatal jaundice were treated promptly. In the first few months of life, he was found to have delayed neurodevelopment and central hypotonia. He was able to hold his head at 5 months, rolled over at 8 months, walked and had first words at 2 years old. At the age of 9 years and 9 months, an IQ test by Wechsler Intelligence Scale for Children: 4th edition (WISC-IV) revealed that his full-scale IQ was 56, indicating intellectual disability. The patient had possible seizure activity at age 10 described as parasomnias, was found to have intermittent bifrontocentreal rhythmic theta activity, and the spells resolved after valproic acid therapy. His height and weight followed the curve of 50th centile, but his head circumference remained at 97th centile (53.5 cm and 55 cm at 6 and 9 years old, respectively). Physical examination was generally unremarkable except macrocephaly and low-set prominent anti-helical pinnae (Fig 1A). Eye examination, hearing tests, thyroid function tests, chromosomal analysis, and nerve conduction velocity were normal. Both brain CT scans at 4 months and 8 years old and brain MRI at 7 months old showed a slight dolichocephalic cranial shape (cephalic index = 75), without overt structural brain abnormalities (Fig 1B–1D). X-ray analysis of the spine showed no obvious scoliosis at 10-years-old (Fig 1E).

To elucidate the genetic etiology, we performed homozygosity mapping, whole genome array comparative genomic hybridization (CGH), and whole exome sequencing (WES). We identified 83 homozygous variants, which had not been reported as SNPs in dbSNP137 (S1 Table). We then selected only those located within the 63 homozygous regions found by homozygosity mapping (S2 Table). Seven candidate variants (one frameshift and six missense mutations; Table 1) were identified. Of the six missense, five were predicted to be either benign by Polyphen-2 or tolerated by SIFT prediction programs. The remaining variant, c.235G>A; p.V79M of the *Carboxypeptidase E* (*CPE*) gene, was not evolutionarily conserved among diverged species (S1 Fig). We, therefore, decided to further our study on the only candidate truncating mutation, a homozygous one base-pair deletion, c.1193delT (p.Leu398Glnfs*2) in exon 11 of *Kinesin family member 6* (*KIF6*) (NM_001289021.2).

**Table 1:** Seven candidate variants from WES and homozygosity mapping.

#	Chromosome (position)	Gene	Zygosity	Nucleotide change	Amino acid change	Polyphen-2 (score)	SIFT
1	6 (39513453)	*KIF6*	Homozygous	c.1193delT	p.L398QfsX399		
2	4 (166300608)	*CPE*	Homozygous	c.235G>A	p.V79M	Possibly damaging (0.889)	Deleterious
3	6 (41895234)	*BYSL*	Homozygous	c.391C>T	p.R131C	Probably damaging (0.975)	Tolerated
4	7 (44120345)	*POLM*	Homozygous	c.359G>A	p.R120Q	Probably damaging (0.999)	Tolerated
5	7 (64167644)	*ZNF107*	Homozygous	c.962T>G	p.I321S	Probably damaging (0.969)	Tolerated
6	2 (96148317)	*TRIM43B*	Homozygous	c.146C>T	p.P49L	Benign (0.071)	Tolerated
7	5 (140307142)	*PCDHAC1*	Homozygous	c.665T>C	p.I222T	Benign (0.001)	Tolerated

*KIF6* is located on human chromosome 6p21.2 and comprises 23 exons. The 2.4-kb *KIF6* cDNA encodes a canonical N-terminal kinesin motor domain (amino acid positions 3–353) and three coiled-coil regions (amino acid positions 358–385, 457–493, and 633–683), predicted by SMART server [20]. Segregation of the homozygous sequence variant with the disease phenotype was confirmed by Sanger sequencing (Fig 1F) and by restriction fragment length polymorphism (RFLP) analysis of the pedigree (Fig 1G), while his parents and his unaffected brother were heterozygous for the deletion (Fig 1F and 1G). The deletion was not observed in our 1,600 in-house Thai exomes, the 1000 Genome Database, and the ExAC Database. The pedigree combined with the novelty of the mutation in *KIF6* presented here, strongly suggest this C-terminal truncating mutation in KIF6 may be etiologic for neurological developmental defects.

### Generation of *Kif6* mutation in mouse

**Fig 2:**
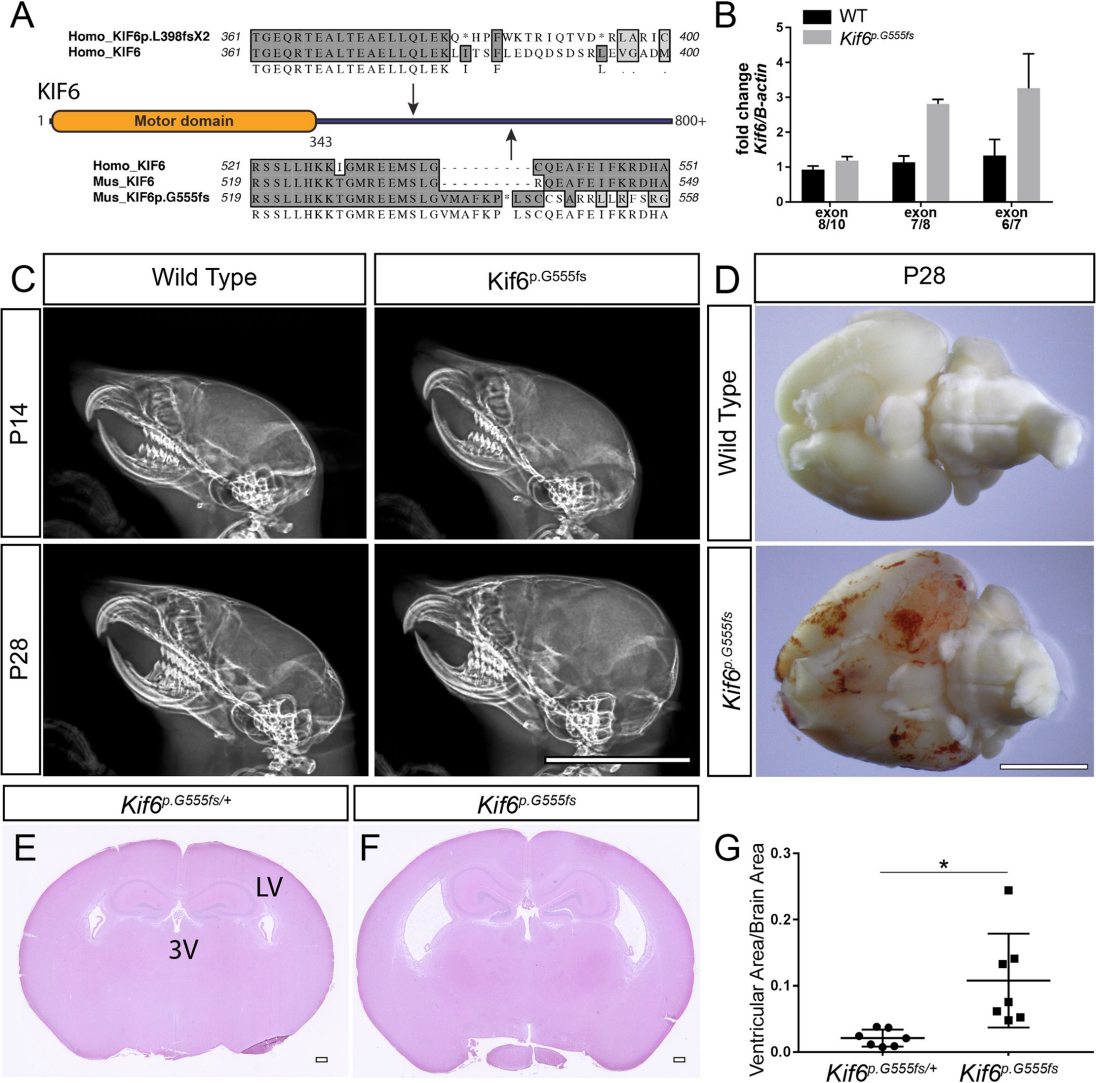
*Kif6* ^*p*.*G555fs*^ mutant mice display progressive hydrocephaly. (A) Schematic of the non-sense mutation in the patient (*KIF6*^p.L398fsX2^) and the mouse mutation (*Kif6*^*p*.*G555fs*^), both predicted to truncate the C-terminal domain of KIF6 protein. (B) qRT-PCR analyses of fold change of *Kif6* expression using cDNA libraries derived from lateral ventricles from WT (black bars) and *Kif6*^*p*.*G555fs*^ (gray bars) mutant mice. (C) Lateral X-rays of mouse cranium at P14 and P28 showing the progressive cranial expansion in *Kif6*^*p*.*G55*^ homozygous mutant mice. (D) Ventral view of whole mouse P28 brain to highlight hemorrhaging and slight enlargement of total brain size in *Kif6*^*p*.*G55*^ homozygous mutant mice. (E, F) H&E stained coronal sections of the mouse brain (P14), showing dilation of the lateral (LV) and third (3V) ventricles in *Kif6*^*p*.*G55*^ homozygous mutant mice (F). (G) Quantitation of ventricular area over total brain area in *Kif6*^*p*.*G55*^ homozygous mutant mice and heterozygous littermate controls (n = 7 mice per genotype; two-tailed t-test; p = 0.0173). Scale bars: 1cm in (C); 5mm in (D); and 300 μm in (F, F).

To test the functional consequence of the C-terminal truncating p.L398fsX2 mutation (Fig 1H), we generated an analogous frameshift mutation in exon14 of the mouse *Kif6* (ENSMUST00000162854) gene, which is ~150bp downstream of the frameshift mutation found in the patient (Fig 2A). After backcross of founder mice to the C57B6/J strain, we identified a nonsense allele with scarless insertion (c.1665ins) of a 3-stop donor cassette -providing integration of an ochre termination codon in all three reading frames into the endogenous *Kif6* locus (S2 Fig). This endonuclease-mediated insertional frameshift mutation (*Kif6*^*em1Rgray*^) is predicted to truncate the C-terminal cargo-binding domain of the kinesin motor protein (p.G555+6fs). This novel mutant allele of *Kif6* (hereafter called *Kif6*^*p*.*G555fs*^) is predicted to encode a C-terminal truncated KIF6 protein 168 amino acids longer than is predicted for the human p.L398fsX2 variant (Fig 2A). Real time qualitative-PCR analysis of several *Kif6* exon-exon boundaries found no evidence for non-sense mediated decay in *Kif6*^*p*.*G555fs*^ mutant mice (Fig 2B).

### Hydrocephalus in *Kif6*^*p*.*G555fs*^ mouse

Intercrossing *Kif6*^*p*.*G555fs*/+^ heterozygous animals gave offspring with the expected Mendelian ratios, with typical appearance at birth. However, beginning at postnatal day (P)14-onwards, 100% (n = 7) of *Kif6*^*p*.*G555fs*^ homozygous mutant mice displayed classic indications of hydrocephalus including doming of the cranium (Fig 2C), a hunched appearance, and with decreased open field activity. We observed apparent megalencephaly and hemorrhaging in older (P21-P28) *Kif6*^*p*.*G555fs*^ mutant brains (Fig 2D), which likely results from increased intracranial pressure and swelling of the ventricles causing damage to the neural tissue against the cranium. At P14, the body weights were not significantly decreased in *Kif6*^*p*.*G555fs*^ mutants (5.8±1.3 (g)rams) compared with littermate controls (7.0±1.2g) (n *= 5*/genotype; *p* = 0.17). However, at P28 mutant mice showed decreased weight on average (12.67±1.53 g) compared to littermate controls (15.33±1.15g), although this trend was not statistically significant (n = 3/genotype; *p* = 0.07). At P28, extracted whole brain sizes appear to be larger in *Kif6*^*p*.*G555fs*^ mutants compared to non-mutant littermate controls (Fig 2D). Due to increased morbidity in these mutant animals no experimental observations were made past P28.

To determine whether a more N-terminal truncated *Kif6* mutation would result in a more severe hydrocephalus phenotype, we isolated a conditional-ready *Kif6* allele, where exon 4 is flanked by LoxP sites (*Kif6*^*tm1c*^) (KOMP repository, see Methods and Materials). Recombination of the *Kif6*^*tm1c*^ allele is predicted to generate a frameshift mutation, which should generate a severely truncated, 89 amino acid, KIF6 protein (p.G83E+6fs) with a non-functional N-terminal motor domain. We generated a whole body conditional knockout by crossing the *Kif6*^*tm1c*^ mouse to the *CMV-Cre* deleter mouse [21]. We observed postnatal-onset, hydrocephalus in *CMV-Cre; Kif6*^*tm1c/tm1c*^ conditional mutant mice (n = 12) analogous to our observations in *Kif6*^*p*.*G555fs*^ mutant mice (S3 Fig). Interestingly, we find no evidence of non-sense mediated decay in these mutant mice despite the generation of an early premature termination codon (Fig 2B). Because the onset and progression of hydrocephalus was equivalent comparing the whole-body conditional *CMV-Cre;Kif6*^*tm1c/tm1c*^ and *Kif6*^*p*.*G555fs*^ mutant mice strains we suggest that any KIF6 protein encoded by these mutant mouse strains is likely non-functional. Given its relevance to the human mutation, the majority of experiments were all done using the *p*.*G555fs* allele.

Mouse brains were analyzed histologically by hematoxylin and eosin (H&E) stained coronal sections. Our analysis of coronal sectioned brain at P14 failed to find significance when comparing the total area in section (499.2+39.9μm (Control) vs. 552.5+50.8μm (*Kif6*^*p*.*G555fs*^); n = 7/genotype; *p* = 0.42). However, lateral and third ventricles (LV and 3V respectively) were obviously enlarged in both *Kif6*^*p*.*G555fs*^ and *CMV-Cre;Kif6*^*tm1c/tm1c*^ mutant mice (Fig 2F and S3 Fig). Quantitation of LV area normalized to total brain area confirmed ventricular expansion in *Kif6*^*p*.*G555fs*^ (n = 7/genotype; *p*≤0.05; Fig 2G) and in *CMV-Cre;Kif6*^*tm1c/tm1c*^ mutant animals (n = 5/genotype; *p*≤0.05; S3 Fig). No obvious defects of the cortex or dysmorphology of other regions of the brain were apparent in these mice (Fig 2E and 2F and S3 Fig). Together these data suggest that *Kif6* mutant mice display postnatal-onset, progressive hydrocephalus, without obvious overgrowth of neural cortex.

### *Kif6* is expressed specifically in the ECs of the mouse brain

**Fig 3:**
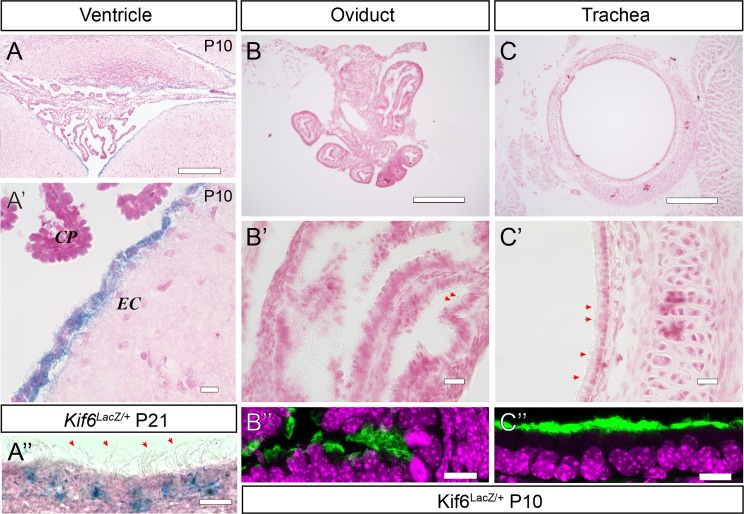
*Kif6-LacZ* expression is specific to the ependymal cells. (A-C', B”-C”) Representative LacZ staining in a variety of multiciliated tissues from P10 and P21 (A”) *Kif6-LacZ*^*tm1b/+*^ transgenic mice. (A, A') Coronal section at the 4^th^ ventricle showing specific *LacZ* expression in the ependymal cell (EC) layer and stark lack of expression in the choroid plexus (CP) or surrounding neuronal tissues. (B-C’) Sectioned oviduct and trachea tissue shows no *LacZ* expression, despite the presence of tufts of cilia observed under oblique lighting (red arrows B’, C’). Immunofluorescence shows acetylated tubulin labeling of ciliary axonemes in adjacent sections in oviduct which has supercoiled at this time point (B”) and in trachea (C”) at P10. LacZ staining labels the EC cells projecting tufts of cilia observed by oblique lighting (A”). Scale bars: 300μm in (A-C); 20μm in (A'-C'); and 10μm in (A”-C”).

To determine the endogenous expression patterns of *Kif6* in the mouse, we also isolated a *Kif6*-*LacZ* reporter mouse (*Kif6-LacZ*^*tm1b*^) (KOMP repository, see Methods and Materials). Hemizygous *Kif6-LacZ*^*tm1b/+*^ mice appeared unremarkable and exhibited no evidence of hydrocephalus. Intercrosses of *Kif6-LacZ*^*tm1b/+*^ hemizygous mice failed to generate litters with *Kif6-LacZ*^*tm1b/tm1b*^ homozygous mice, suggesting that the homozygosity of the *lacZ* expressing allele is embryonic lethal (0/13 *Kif6-LacZ*^*tm1b/tm1b*^ homozygous mutant mice from 3 independent litters). At P10 and P21, *Kif6-LacZ*^*tm1b/+*^ transgenic mice showed *lacZ* expression in the ECs of the ventricular system (red arrows; Fig 3A”) and the central canal (S4 Fig). However, no *lacZ* expression was detected in the choroid plexus or in other regions of the brain (Fig 3A and 3A’ and S4 Fig), with the exception of a small population of cells, possibly the hypothalamic nuclei, flanking the third ventricle (arrows, S4 Fig). Interestingly at P10, other multi-ciliated tissues such as the oviduct or trachea were not labeled in these transgenic mice, despite the clear presence of cilia observed by oblique lighting (red arrows Fig 3B’ and 3C’) as well as by IF using acetylated-tubulin to label axonemes in adjacent sections of these tissues (Fig 3B” and 3C”). No obvious changes to oviduct or trachea cilia were observed in *Kif6* ^*p*.*G555fs*^ mutant mice at P21 (S5 Fig), suggesting that *Kif6* expression and function are tightly restricted to the multiciliated ECs in mouse. Taken together these data suggested a cellular mechanism centered on defective ECs underlying the development of hydrocephalus in *Kif6* mutant mice.

### Loss of Cilia in *Kif6* mutant mice

**Fig 4:**
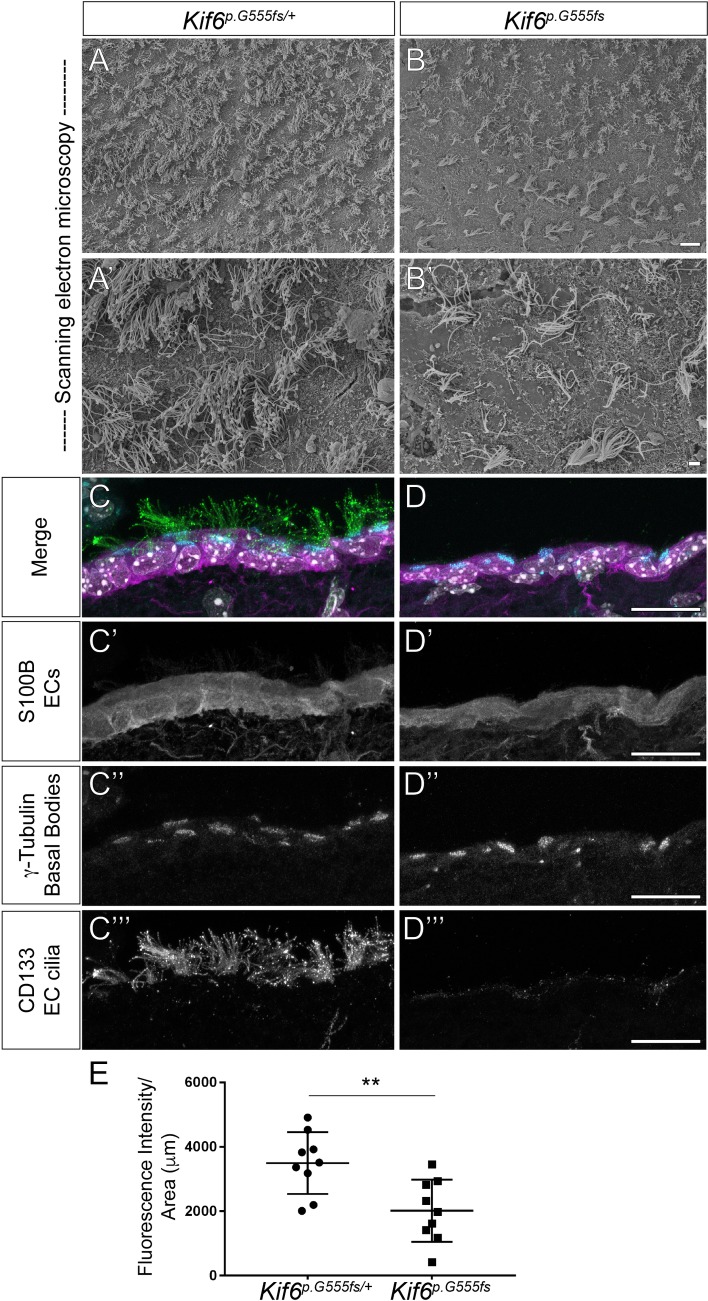
*Kif6* mutant mice have defects in formation of ependymal cell cilia. (A-B') Scanning electron microscopy of the lateral ventricular wall (*en face* view) in *Kif6*^*p*.*G55*^ homozygous mutant mice and heterozygous littermate controls at P21, demonstrating a reduction in the number and density of EC cilia tufts in mutant ventricles (B, B’). (C-D‴) Immunofluorescence of the wild-type (C-C”‘) and *Kif6*^*p*.*G55*^ homozygous mutant (D-D”‘) mice at P21. (C-D) Three color merge of (C', D') αS100B (ependymal cell marker; magenta) channel; (C'', D'') α–γ-tubulin (basal bodies; cyan) channel; and (C‴, D‴) αCD133 (EC axoneme marker, Prominin-1; green). (C', D') αS100B staining showing no alterations of ependymal cell specification between *Kif6*^*p*.*G55*^ homozygous mutant and WT mice. (C'', D'') α–γ-tubulin staining showing typical basal body positioning at the apical surface of ECs in both *Kif6*^*p*.*G55*^ homozygous mutant and WT mice. (C‴, D‴) αCD133 staining reveals a marked of EC cilia projecting into the ventricular lumen in *Kif6*^*p*.*G555fs*^ homozygous mutant mice compared to WT mice. (E) Quantitation of fluorescent intensity of the CD133 channel (EC axonemes). Scale bars: 20μM in (A, B); 2 μM in (A', B'); and 20 μM in (C-D‴).

Defects of EC cilia are known to cause hydrocephalus in mouse [8]. To assay this we first utilized scanning electron microscopy (SEM) to directly visualize the LV *en face*. Heterozygous *Kif6*^*p*.*G555fs/+*^ mice displayed a high-density of regularly spaced EC multiciliated tufts along the LV surface (Fig 4A and 4A’), typical at P21 in mouse development [22]. In contrast, homozygous *Kif6*^*p*.*G555fs*^ mutant mice displayed a marked reduction of multiciliated tufts across the LV wall (*p* = 0.029; Avg. # ECs /section_het_ = 225; Avg. # ECs /section_mutant_ = 129.3), coupled with a reduction in the density of ciliary axonemes within these ciliary tufts (Fig 3B and 3B’). This phenotype was even more obvious at P28 in Kif6 mutant mice (*p* = 0.001; Avg. # ECs /section_het_ = 146; Avg. # ECs /section_mutant_ = 0) (S6 Fig). Together, these data suggested that hydrocephalus may result from either a reduction in EC differentiation and/or defects in EC cilia during postnatal development.

To address the differentiation status of the ECs, we utilized IF in coronal sectioned brain tissues to image known proteins components of the EC and their cilia. At P21, we observed the expression of the ependymal cell-marker S100B [22] throughout the epithelium lining luminal surface of the ventricles, as well as, the presence of apically localized γ-tubulin-positive basal bodies within these ECs in both WT (Fig 4C and 4C'') and *Kif6*^*p*.*G555fs*^ mutant mice (Fig 4D and 4D''). Conversely, we observed a obvious reduction in the density of CD133-positive EC axonemes [23] extending into the ventricular lumen in *Kif6*^*p*.*G555fs*^ mutant mice (Fig 4C and 4C‴), compared with WT (Fig 4D and 4D‴). Quantitation of binned mean fluorescence intensity from of CD133-positive axonemes confirmed a severe reduction of EC axonemes in Kif6 mutant mice (n = 9 mice/genotype, *p*≤0.001) (Fig 4E).

In order to address whether *Kif6* is required for EC cilia formation or ciliogenesis, we performed IF at P14, the time point at which the ECs are fully ciliated across the ventricular system in mouse [22]. We observe significant alterations in the extension of EC ciliary axonemes (CD133-positive) (n = 5 mice/genotype, *p*≤0.01), without an alterations in the specification of ECs or their apical polarity (S7 Fig). The reduction of EC cilia was also observed at both P14 and P21 in *Kif6*^*p*.*G555fs*^ mutant mice using an acetylated tubulin antibody, which labels both primary and motile cilia as well as neuronal cell types in the cortex (S8 Fig). Taken together, these results suggest that the onset of hydrocephalus in *Kif6* mutant mice is primarily due to a general defect of cilia formation (ciliogenesis) and not the result of defects in differentiation of the ECs or a loss of EC cilia as the result of the onset of hydrocephalus.

Alterations of the motility or synchronicity of EC cilia beating are known to cause hydrocephaly in mouse [8]. In order to address if *Kif6* is required for normal EC cilia beating we performed live *ex vivo* imaging of lateral wall explants taken from both *Kif6* mutant and littermate control mice. We consistently observed multiple tufts of EC cilia in WT animals that beating in a synchronous fashion at P21, with obvious flow generation, demonstrated by the movement of fortuitous particles within the media (S1 Movie). As expected, we observed an obvious reduction in formation of EC cilia coupled with reduced particle flow in explants from *Kif6* mutant mice (S2 Movie). Interestingly, on the rare occasions where we did observed EC cilia in these mutant mice, the motility of these EC cilia appeared typical. These results in conjunction with our IF and SEM analysis (Fig 4, S6–S8 Figs) suggest that the onset of progressive hydrocephaly in *Kif6* mutant mice is the result of defective ciliogenesis of ECs leading to a reduction in near-wall CSF flow.

### Ventricular dilation and Reduced EC cilia in *kif6* mutant zebrafish

**Fig 5:**
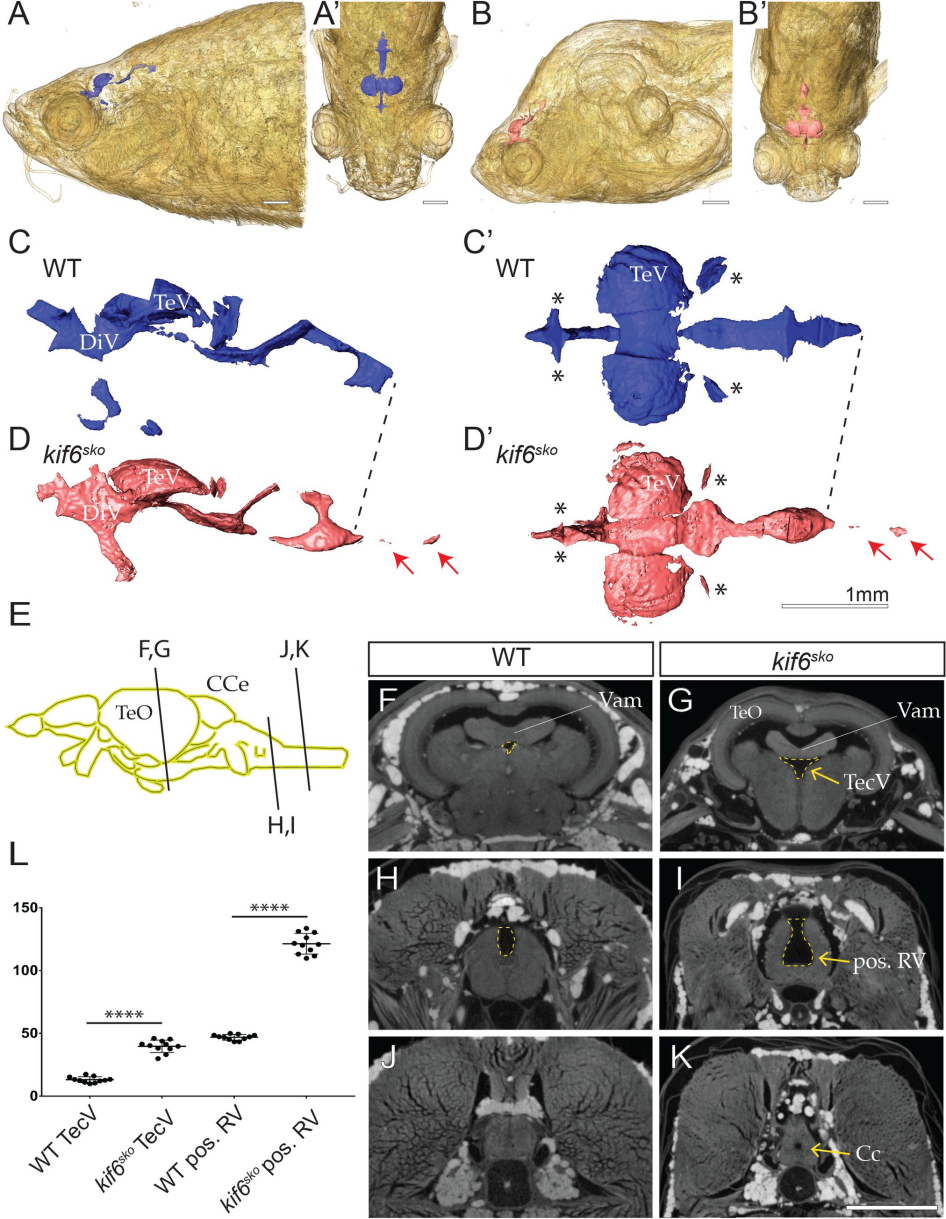
*kif6* mutant zebrafish display dilation of the ventricular system. (A-B’) 3D-reconstruction of representative iodine-contrasted μCT dataset from WT (A-A’) and *kif6*^*sko*^ mutant zebrafish at 90dpf. (C-D’) 3D-reconstruction and segmentation of virtual endocasting of ventricular system in WT (blue; C-C’) and *kif6*^*sko*^ mutant (red; D-D’) zebrafish from datasets in A-B’ demonstrated morphological alterations of the ventricular system including dilation of the central canal (red arrows; D-D’) and stenosis of small ventricles (asterisks, D-D’). (E) Schematic of adult zebrafish brain highlighting the relative transverse optical section of the zebrafish brain in WT (F, H, J) and *kif6*^*sko*^ homozygous mutant (G, I, K) zebrafish brain at 90dpf. (F, G) The medial region of the TeO showing the medial TecV (yellow dashed line) which is dilated in *kif6*^*sko*^ mutant fish (G) compared to age-matched WT (F). (H, I) Sectioning at the region of the medulla oblongata posterior to the lobus facialis showing dysmorphogenesis and deepening of the posterior RV (yellow dashed line) in *kif6*^*sko*^ mutants (I) compared with WT (H) zebrafish. (J, K) Spinal cord sectioning showing dilation of the central canal in *kif6*^*sko*^ mutant (K) compared to WT (J) zebrafish. (L) Quantitation of the areas (yellow dashed line) of the TecV and the RV posterior to the lobus facialis (pos. RV) in WT and *kif6*^*sko*^ mutant zebrafish, highlighting a consistent dilation in *kif6*^*sko*^ mutants (n = 11 sections/genotype; two-tailed t-test; ****, p<0.0001). Scale Bars: 1mm. *DiV—*diencephalic ventricle; *TecV-tectal ventricle TeO-tectum opticum; CCe-corpus cerebelli; RV- rhombencephalic ventricle; Vam—medial division of valvula cerebelli; and Cc-central canal*.

Defects in EC cilia in the central canal leads to impaired CSF flow and hydrocephalus in zebrafish embryos [24]. Our previous studies in *kif6*^*sko*^ mutant zebrafish observed late-onset scoliosis in larval zebrafish, without defects of CSF flow, EC cilia in the central canal, or hydrocephalus during early embryonic development [19]. However, in contrast to the defined period of EC development in the mouse, zebrafish demonstrate continuous differentiation of ECs within the ventricular system throughout adult development [25]. Interestingly, recent studies in a variety mutant zebrafish demonstrate that reduced CSF flow, ventricular dilation, and loss of EC cilia may underlie the onset late-onset scoliosis in larval zebrafish [26]. In order to determine if *kif6* mutant zebrafish might also display changes in the ventricular system in adults, we used iodine contrast-enhanced, micro computed tomography (μCT) [27] to generate high-resolution (5 μm voxel size) images of aged matched, 3-month-old WT and *kif6*^*sko*^ homozygous mutant zebrafish (Fig 5A and 5B’). After reconstruction and alignment of 3D tomographic datasets in the coronal plane, we utilized visualization software (Avizo Lite v.9.5) for 3D-reconstruction and segmentation of a virtual endocast to represent the ventricular volume in wild-type (WT) and *kif6*^*sko*^ mutant zebrafish (Fig 5A–5D’). Analysis of the ventricles in the endocast of the adult zebrafish brain highlighted several dysmorphophic regions in *kif6*^*sko*^ mutants including dilation of the diencephalic ventricle (DiV) (Fig 5D) and dilation of regions of the central canal (red arrows; Fig 5D and 5D’). At the same time, we observe that while some ventricles patent in wild-type zebrafish, were completely obstructed or less open in *kif6*^*sko*^ mutants (asterisks; Fig 5C’ and 5D’).

To further describe and quantify alterations of the ventricular system we utilized individual transverse optical slices from these contrast-enhanced μCT datasets, using stereotyped landmarks of the zebrafish brain and spinal cord to compare equivalent axial sections. At distinct axial levels of the brain (Fig 5E), we observed consistent dilation of the ventricular system and central canal in *kif6*^*sko*^ homozygous mutant zebrafish (yellow arrows; Fig 5G, 5I and 5K), compared to the stereotyped anatomy described for the adult zebrafish brain (Fig 5F, 5H and 5J) [28]. Multiple regions of *kif6*^*sko*^ mutant zebrafish brain were found to be structurally abnormal in *kif6*^*sko*^ mutants compared to WT zebrafish (S3, S4 Movies). We next quantified the areas of two anatomically distinctive ventricles in our tomographic datasets: (i) the medial tectal ventricle (TecV) at the medial division of valvula cerebelli (Vam) (Fig 5F and 5G) and (ii) a region of the rhombencephalic ventricle (RV) just posterior to the lobus facialis (Fig 5H and 5I) [28]. We observed a significant increase in the area (dashed yellow line, Fig 5F–5I) of the medial TecV and the posterior RV in *kif6*^*sko*^ mutant zebrafish comparing several optical sections from independent aged-matched zebrafish (n = 3 fish/genotype; *p*<0.0001). The central canal was also clearly dilated in *kif6*^*sko*^ mutant zebrafish (yellow arrow, Fig 5K). However, we were unable to reliably quantify this area in WT samples at the current imaging resolution. Our previous observations in *kif6*^*sko*^ mutant zebrafish embryos failed to find phenotypes that are characteristic of cilia defects, such as hydrocephalus, *situs inversus*, or kidney cysts [19]. Moreover, we observed normal development and function of motile EC cilia within the central canal in embryonic mutant zebrafish [19]. These data, coupled with our new observations of ventricular dilation in adult *kif6* mutants (Fig 5), suggest that Kif6 is required for the post-embryonic, robust development of the EC cilia within the ventricles of the brain as was reported in other zebrafish mutants displaying similar late-onset scoliosis, as observed in *kif6* mutant zebrafish [19, 26].

**Fig 6:**
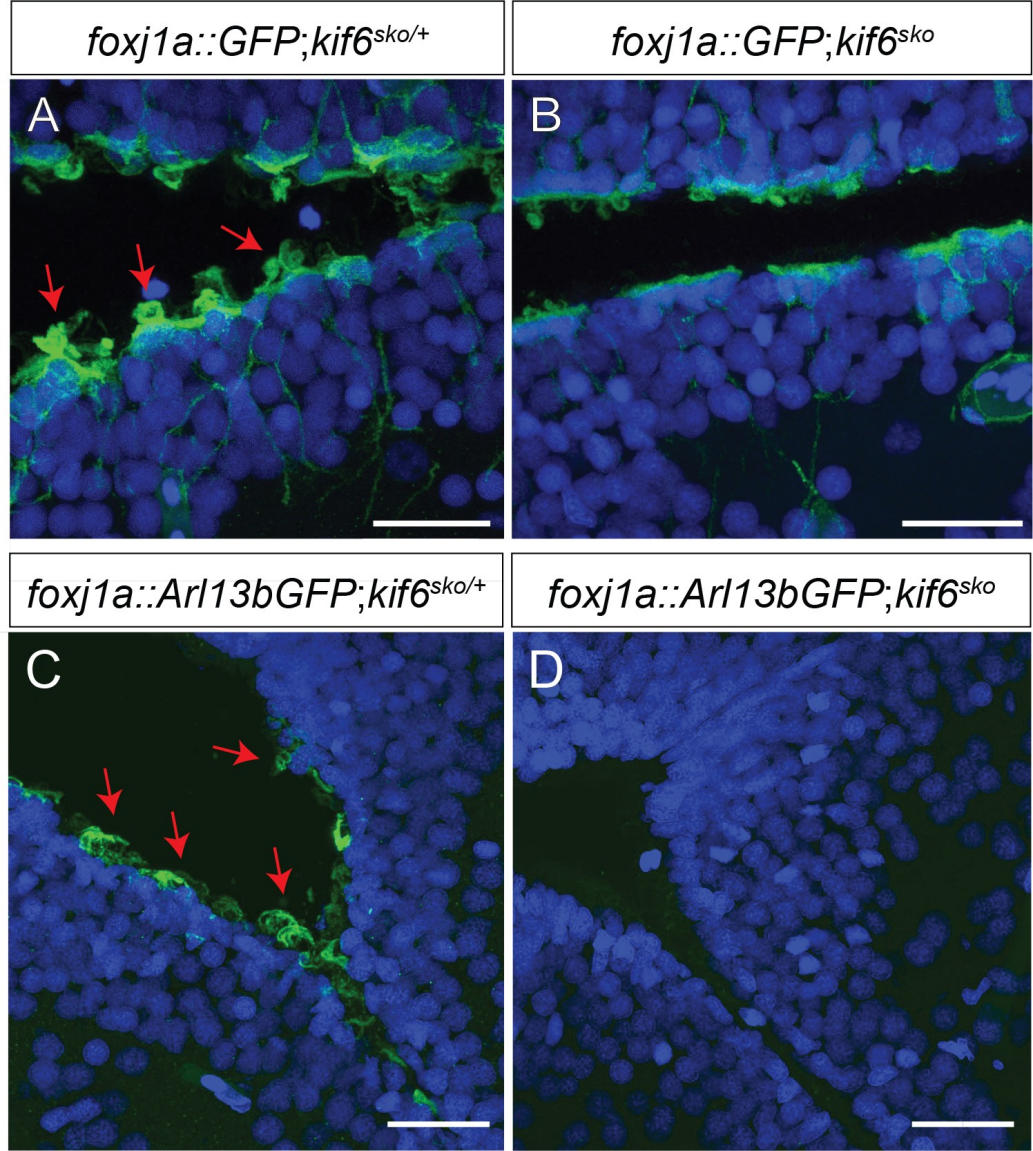
*kif6* mutant zebrafish display defects in the formation of the ependymal cell cilia in the brain. (A-D) Immunofluorescence of *Tg[foxj1a*::*GFP]* (A, B) and *Tg[foxj1a*::*Arl13bGFP]* transgenic zebrafish in both heterozygous *kif6*^*sko/+*^ (A, C) and homozyogus *kif6*^*sko*^ mutant (B, D) zebrafish backgrounds assayed with αGFP (green) and DAPI (nuclei, blue). The *Tg[foxj1a*::*GFP]* transgene demonstrates that GFP positive ECs are present in both genotypes. In contrast, *kif6*^*sko/+*^ heterozygous zebrafish display numerous apical tufts of cilia (red arrows; A) projecting into the ventricle lumen, which are markedly reduced in *kif6*^*sko*^ mutant zebrafish (B). Similar results were observed with the *Tg[foxj1a*::*Arl13bGFP]* transgene, showing a obvious reduction in Arl13b-GFP positive EC axonemes in *kif6*^*sko*^ mutant zebrafish (D), which are robustly labeled in *kif6*^*sko/+*^ heterozygous fish (C). Scale Bars: 20μm.

In order to assay whether EC cilia were affected during adult development in zebrafish, we isolated a stable transgenic allele, *Tg(Foxj1a*:*GFP)*^*dp1*^ previously reported to label multiciliated *Foxj1a*-positive multi-ciliated cells (MCCs), including ECs, with cytoplasmic EGFP in zebrafish [26]. Using this transgenic approach, we observed no differences in the specification of *Foxj1a*:*GFP*-positive ECs comparing adult (90dpf) heterozygous *kif6*^*sko/+*^ phenotypically wild-type and homozygous *kif6*^*sko*^ mutant fish (Fig 6A and 6B). Cytoplasmic GFP can freely diffuse through the transition zone of cilia and label the axoneme [29]. As such, we were also able to observe GFP-filled EC axonemes projecting into the ventricular lumen in *Tg(Foxj1a*:*GFP)*^*dp1*^; *kif6*^*sko/+*^ heterozygous fish (red arrowheads; Fig 6A). In contrast, these GFP-filled EC axonemes were reduced or absent in *Tg[Foxj1a*:*GFP]* ^*dp1*^; *kif6*^*sko*^ mutant fish (Fig 6B). To further support our model that EC cilia were affected in *kif6* mutant zebrafish, we generated a novel-transgenic fish line *Tg(Foxj1a*:*Arl13b-GFP)*^*dp22*^ to allow for the direct fluorescent-labeling of ciliary axonemes with mouse Arl13b-GFP specifically in *fox1a*-expressing lineages. Analysis of *kif6*^*sko*^*; Tg(Foxj1a*:*Arl13b-GFP)*^*dp22*^ transgenic adult mutant zebrafish (90dpf) demonstrated an obvious reduction in Arl13b-GFP labeled axonemes (Fig 6D), in comparison to robust labeling of EC cilia in heterozygous *kif6*^*sko/+*^*;Tg(Foxj1a*:*Arl13b-GFP)*^*dp22*^ sibling fish (Fig 6C). Furthermore, SEM imaging of the telencephalic and rhombencephalic ventricles in *kif6*^*sko*^ mutant zebrafish demonstrated: (i) ventricular dilation (dashed red line, S9 Fig) and (ii) an obvious reduction in EC cilia in adult zebrafish (*p* = 0.014; Avg. # ECs /section_het_ = 37.5; Avg. # ECs /section_mutant_ = 1) (red arrowheads, S9 Fig). Akin to our observations in *Kif6* mutant mice trachea, we did not observe defects of other multiciliated tissues in *kif6*^*sko*^ zebrafish mutants, such as the nasal cilia (S5 Fig). Together, these data suggest that Kif6 functions specifically in the formation of EC cilia, in regulation of ventricular homeostasis during adult development in zebrafish.

Fluorescently-tagged Kif6 (Kif6-GFP) localizes to the basal body of Kuper’s vesicle motile cilia in zebrafish [30]. Indeed our own attempts to visualize Kif6-EGFP by microinjection demonstrate that Kif6-EGFP localizes to microtubule-rich spindle poles and mitotic spindle during cell divisions in the rapidly dividing blastomeres of the early embryo (S10 Fig). Our attempts to drive Kif6-EGFP within the ECs using the *foxj1a* promoter in transgenic zebrafish have failed for this purpose, whereas clear signal was obtained for similar *foxj1a-*driven GFP and Arl13bGFP transgenic constructs (Fig 6). Unfortunately, IF and Westerns using several commercially-available KIF6 antibodies were unsuccessful to report on endogenous KIF6 in both mouse and zebrafish tissues (S11 Fig).

**Fig 7:**
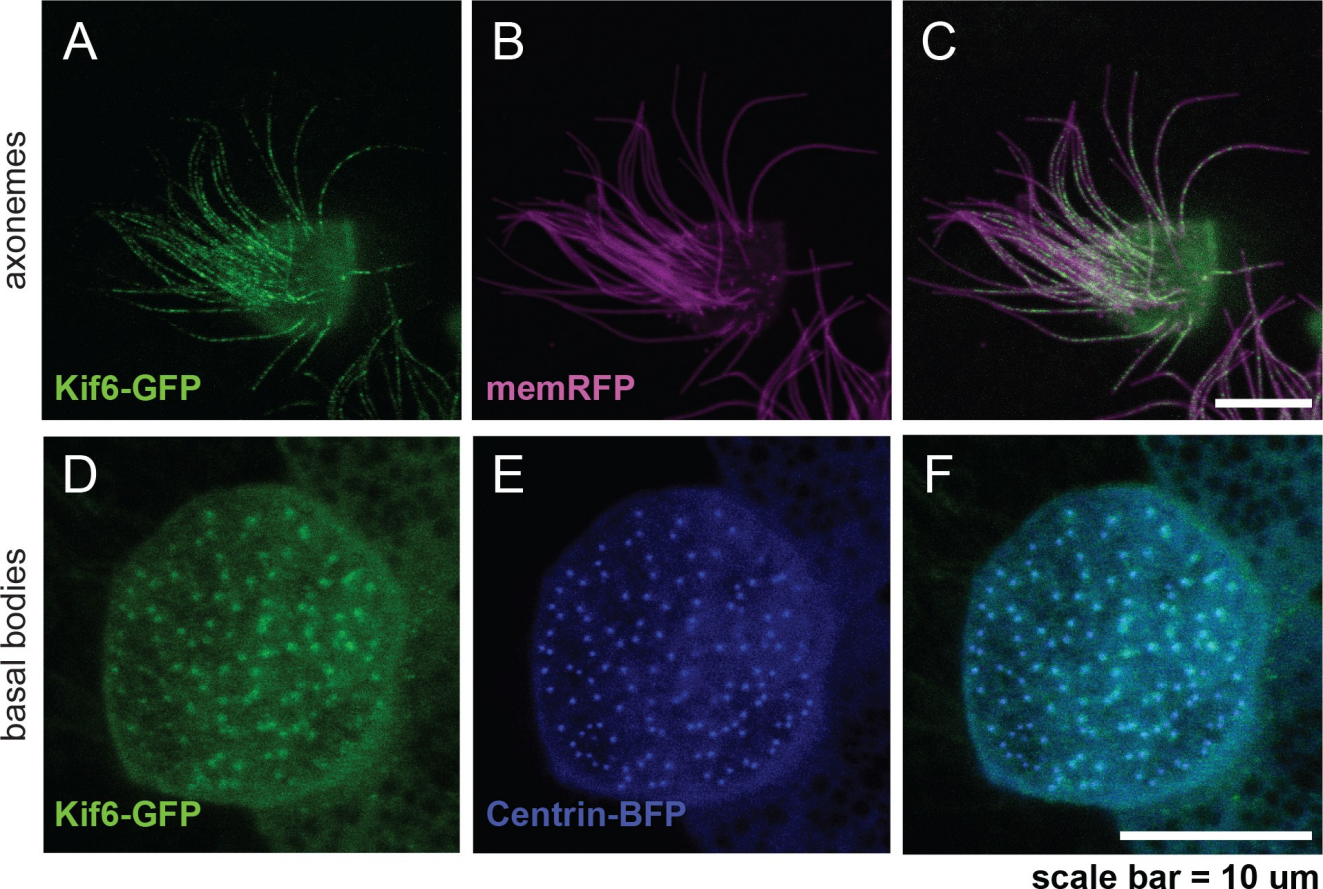
Kif6-GFP localizes to the basal bodies and axonemes of *Xenopus* multi-ciliated cells. Confocal imaging of the mucocilated *Xenopus laevis* epidermis demonstrating consistent Kif6-GFP localization within the axonemes (green; A, C) and at the basal bodies (green; D, F). Expression of pan-membrane-RFP marker (magenta; B, C) to co-label the axonemes and Centrin-BFP (blue; E, F) to co-label the basal bodies. Scale Bars: 10μm.

For these reasons, we turned to the muco-ciliated *Xenopus laevis* epidermis in order to address Kif6 localization in an analogous MCCs lineage. The *Xenopus* mucociliary epithelium is analogous to the airway epithelium of the mammalian trachea [31]. Importantly, this system provides a robust model system for both genetic analysis [32] and robust visualization of fluorescently-tagged proteins in MCCs in vivo [33, 34]. By microinjection of synthetic *Xenopus Kif6-GFP* RNA, we observed Kif6-GFP localization at the basal bodies of MCCs (Fig 7D and 7F) and co-localized with a basal body marker; Centrin-BFP [35](Fig 7E and 7F). Similarly, we observed Kif6-GFP puncta within the axoneme (co-labeled with pan-membrane bound RFP) (Fig 7A–7C). Interestingly, similar punctate localization at the basal bodies and within the axoneme has been previously been shown in Xenopus MCCs using various fluorescently-tagged IFT proteins [36]. Together these data suggest a model in which Kif6 may act as a component of IFT trafficking uniquely and specifically in EC cilia, which is dispensable for the function of other MCCs in mouse and zebrafish.

## Discussion

This study demonstrates the importance of *KIF6* for EC cilia formation and homeostasis of the ventricular system in vertebrates, and potentially implicates a novel locus for understanding neurological defects in humans. This is supported by several lines of evidence including the discovery of a novel nonsense-mutation of *KIF6* in a child with intellectual disability and megalencephaly and underscored by functional analysis in both mouse and zebrafish *Kif6* mutant models (Table 2).

**Table 2:** KIF6 mutations discussed in this paper.

ID/Allele	Species	Nucleotide change	Amino acid change	Phenotype reported	Reference
Patient	Human	c.1193delT	p.L398QfsX399	Delayed neurodevelopment and central hypotonia, neurological defects, and intellectual disability.	this work
*Kif6*^*p*.*G555fs*^	Mouse	c.1665ins	p.G555+6fs	Severe progressive hydrocephalus, loss of ependymal cell cilia	this work
*CMV-Cre;Kif6*^*tm1c*^	Mouse	Whole-body conditional deletion of exon 4	p.E83+39fs	Severe progressive hydrocephalus, loss of ependymal cell cilia	this work
*Kif6Δ*^*3/*^*Δ*^*3*^	Mouse	c.177-251_del	p.Lys59Asn, Phe60_Ser84_del	None reported	[37]
*kif6*^*sko*^	Zebrafish	c.205C>A	p.Tyr53X	Larval onset scoliosis, hydrocephalus, loss of ependymal cilia	[19], this work

We identified a homozygous *KIF6* c.1193delT mutation in a child with macrocephaly and cognitive impairment that segregated with this phenotype in his family, and leads to a loss of the C-terminal second and third coiled-coil regions which are important for dimerization and cargo selectivity of kinesin motors [13]. We engineered an analogous, C-terminal truncating mutation of *KIF6* in mouse, which displays severe hydrocephalus and defects of EC cilia providing strong evidence for pathogenicity of the mutation in the child. Other than the case described here, no prior mutation directly attributed to human disease has been described for *KIF6*. Taken together, the clinical data reported here suggest that biallelic mutations in *KIF6* may underlie some unexplained intellectual disability and neurological developmental defects. Future analyses of *KIF6* mutations in these patient groups are warranted.

In addition, our analyses of several independent loss-of-function *Kif6* mutant animal models found no evidence of obvious heart abnormalities to explain the prior association of the common variant *KIF6* p.W719R in some[17], but not all [18], studies of coronary heart disease in humans. Because expressed sequence tag clones of *KIF6* have not been reported from cDNAs libraries derived from human heart or vascular tissues (UniGene 1956991—Hs.588202), any possible functional effects of KIF6 on heart function remains unexplained. However, detailed analysis of coronary function was not explored in our models, therefore it is possible that subtle defects may be present.

Previous reports of an ENU-derived *Kif6* splice acceptor site mutant mouse strain, predicted to delete the 3rd exon of *KIF6* (*Kif6Δ*^*3/*^*Δ*^*3*^), also did not show cardiac or lipid abnormalities [37]. Of note this mutant mouse was also not reported to have hydrocephalus. Our analysis shows that the loss of exon 3 in *Kif6Δ*^*3/*^*Δ*^*3*^ mutant mouse generates an inframe deletion of only 25 amino acids in the N-terminal motor domain of the KIF6 protein, otherwise generating a mostly full-length KIF6 protein (Table 2). In contrast, here we report two novel *Kif6* mutant mice: (i) a C-terminal *Kif6*^*p*.*G555fs/p*.*G555fs*^ deletion mutant, predicted to truncate 248 amino acids of the C-terminal domain, which are important for cargo binding in Kinesin motor proteins [13]; and (ii) a conditional *CMV-Cre;Kif6*^*tm1c/tm1c*^ mutant which recombines exon 4 leading to an early frame shift mutation predicted to generate a N-terminal truncated 122 amino acid KIF6 protein (Table 2), both of which display indistinguishable progressive, hydrocephalus with EC ciliogenesis defects. The most parsimonious explanation for the difference in phenotypes in these mutant mice is that the *Kif6Δ*^*3*^ allele encodes a functional KIF6 protein. Analysis of these mutations in trans or quantitative analysis of these kinesin motor proteins *in vitro* is warranted to more fully address these conflicting observations.

There are noticeable differences in the phenotypes among the human, mouse, and zebrafish associated with mutations in *KIF6*. For example, *kif6* mutant zebrafish display post-natal onset scoliosis, mirroring adolescent idiopathic scoliosis (IS) in humans [38]. The formation of IS-like defects in zebrafish has been shown to be the result of a loss of CSF flow, associated with a loss of EC cilia and ventricular dilation during a defined window of larval zebrafish development [26]. Interestingly, we did not observe scoliosis in the *Kif6* mutant mice (S12 Fig), despite being of an appropriate age when IS-like scoliosis can manifest in mouse [39]. Moreover, we do not observe scoliosis in the patient at the age of 10 years, though it is possible that he may yet develop scoliosis during adolescence. The mechanisms behind these differences may reflect distinctions in the functional input of the ventricular system for spine stability amongst teleosts and amniotes.

Furthermore, while we observe a clear role for KIF6 in maintaining the ventricular system in mouse and zebrafish, the patient does not have obvious hydrocephalus. However, his relative macrocephaly and slightly enlarged ventricles by MRI (Fig 1B–1D) may suggest an element of what is commonly referred to as arrested hydrocephalus [40]. Moreover, the contribution of EC cilia beating to bulk CSF flow might be species dependent. For instance, the majority of CSF flow in humans is thought to occur via the generation of a source-sink gradients; partly from the secretion of the choroid plexus and exchanges of the interstitial fluids, coupled with absorption at the arachnoid villi and lymphatics [41]. In contrast, localized or near-wall CSF flow [4], generated by polarized beating of EC cilia, are clearly important for the formation of hydrocephalus in rodents [8]; however, there have been limited evidence of EC cilia defects causing hydrocephalus in humans. Regardless there is growing evidence suggesting that EC cilia dependent CSF flow is crucial for the regulation of brain function and neurogenesis [4], and for adult neural stem cell proliferation [9]. It is possible that a specific loss of EC cilia in humans may only have minor effects on CSF bulk flow and ventricular homeostasis, while causing severe defects of neurogenesis leading to intellectual disability and other neurological disease. It will be important to determine (i) if the loss of KIF6 function during adult development in mouse will lead to a reduction in EC cilia; and (ii) whether the loss of EC cilia function contributes to ventricular dilation and decline of neurological function.

Finally, *KIF6* now joins five other kinesin genes, *KIF1C*, *KIF2A*, *KIF4A*, *KIF5C* and *KIF7* that were previously reported to be associated with neurological abnormalities in humans [42–45]. Here we suggest that *KIF6* has a uniquely specific function in the EC cilia in vertebrates, resulting in both cognitive impairment and macrocephaly in a child with a homozygous one-base pair deletion. Using a cell biological and transgenic approaches in both mouse and zebrafish, we identified specific loss of EC cilia these *Kif6* mutant models suggesting a strong conservation of KIF6 function in ventricular system in vertebrates. Furthermore, we utilized imaging of fluorescently-tagged Kif6 in MCCs of the Xenopus epidermis, which are anatomically and functionally analogous to ECs lining the ventricles of the brain. Using this heterologous system we demonstrated that Kif6-GFP localizes to the basal bodies and as puncta within the ciliary axonemes in these MCCs, which is reminiscent of observations of canonical IFT proteins [36, 46]. This imaging data coupled with our findings of defective ciliogenesis in ECs in both *Kif6* mutant mice and zebrafish suggest a model where KIF6 is acting in concert with one or more anterograde Kinesin-II motors to promote robust ciliogenesis specifically in ECs. Indeed, tissue specific accessory IFT motors have been described in amphid sensory neurons in *C*. *elegans* [47] and zebrafish photoreceptors [48, 49]. Interestingly, a homologue of KIF6, KIF9B, has been shown to be critical for flagellar motility and for the stabilization of the paraflagellar rod structure which tightly abutted the flagella in the protist, *Trypanosoma brucei* [15]. It remains to be determined whether KIF6 has an analogous, structural-functional role in ECs which may provide a unique functional role in these cilia, which is not critical for other analogous MCC lineages such as the trachea or oviduct. The confinement of LacZ expression specifically with EC in the *Kif6-LacZ*^*tm1b*^ gene trap mouse suggest a large part of this specificity of function is simply due to strict regulation of *Kif6* expression to the EC lineages, which would implicate the action of a tightly regulated, EC specific-promoter driving expression of the Kif6 locus. Both models will be important to test in future studies to better understand the critical components and pathways important for EC cilia development.

## Material and methods

### Ethics statement

The collection and use of human DNA samples in this study was approved by the Institutional Review Board Faculty of Medicine, Chulalongkorn University, Bangkok, Thailand (IRB 381/61). All subjects provided written informed consent prior to inclusion in the study.

All animal research was conducted according to federal, state, and institutional guidelines and in accordance with protocols approved by Institutional Animal Care and Use Committees at University of Texas at Austin (AUP-2015-00185; AUP-2015-00187; and AUP-2018-00225).

### Whole exome sequencing (WES)

The patient’s genomic DNA of patient was extracted from peripheral blood leukocyte using AchivePure DNA Blood Kit (5 Prime Inc., Gaithersburg, MD). The sample was sent to Macrogen, Inc. (Seoul, Korea) for whole exome sequencing. The 4 ug of DNA sample was enriched by TruSeq Exome Enrichment Kit and was sequenced onto Hiseq 2000. The raw data per exome was mapped to the human reference genome hg19 using CASAVA v1.7. Variants calling were detected with SAMtools.

### Homozygosity mapping

The sample was sent to Macrogen, Inc. (Seoul, Korea) for genotyping. The DNA sample was genotyped by HumanOmni 2.5-4v1 DNA BeadChip (Illumina) which contain 2,443,177 SNPs. The experiment was performed by the array protocol. PLINK was used to analyze for the homozygous regions.

### Mutation analysis

We performed resequencing of *KIF6* pathogenic region in patient and patient’s family. Primers for the amplification of the candidate variant were designed using Primer 3 software (version 0.4.0). Primers KIF6-1193delT-F 5’-CAGCTTGAACATGGCTGAAA-3’ and KIF6-1193delT-R 5’-TTCTGTAAAGAGGTGGGAACAA-3’were used to amplify. The 20 ul of PCR reaction contained 50–100 ng of genomic DNA, 200 uM of each dNTP, 150 nM of each primer, 1.5 mM MgCl_2_ and 0.5 unit of Taq DNA polymerase (Fermentas Inc., Glen Burnie, MD). The PCR condition was started with 95 ^o^C for 5 min for pre-denaturation following with the 35 cycles of 94 ^o^C for 30 sec, 55 ^o^C for 30 sec and 72 ^o^C for 30 sec. The product size of these primers is 276 bp. For sequencing, PCR products were treated with ExoSAP-IT (USP Corporation, Cleveland, OH), and sent for direct sequencing at Macrogen Inc. (Seoul, Korea). Bi-directional sequencing was done by using KIF6-1193delT F and R primers. Analyses were performed by Sequencher 4.2 (Gene Codes Corporation, Ann Arbor, MI).

### PCR-RFLP

One hundred chromosomes and patient’s trio were genotyped by PCR-RFLP. Primer KIF6 *Mfe*I F 5’-TGGCTTCACTATAAATTTCACTTTGTCAATG-3’ and mutagenic primer KIF6 mutagenic MfeI R 5’-TCCTGGTCTTCCAAAAAGGATGCAAT-3’were used to amplify KIF6 T-deletion. The 20 ul of PCR reaction contained 50–100 ng of genomic DNA, 200 uM of each dNTP, 150 nM of each primer, 1.5 mM MgCl_2_ and 0.5 unit of Taq DNA polymerase (Fermentas Inc., Glen Burnie, MD). The PCR condition was started with 95 C for 5 min for pre-denaturation following with the 35 cycles of 94 C for 30 sec, 60 C for 30 sec and 72 C for 30 sec. The product size of these primers is around 223 bp. The PCR product was incubated with 10U of Mfe-HF (New England Biolabs, Ipswich, MA) at 37 C overnight. Three percent of agarose gel electrophoresis was used to detect the different cut sizes of PCR product. A 196 bp and 26 bp bands were present in one base deletion sample.

### Mice

All mouse studies and procedures were approved by the Animal Studies Committee at the University of Texas at Austin (AUP-2015-00185). The *Kif6*^*p*.*G555fs*^ mutant mouse were developed using CRISPR-Cas9-mediated genome editing. Using the CHOP-CHOP online tool [50], we identified a suitable 20-nucleotide site (GGAGATGTCACTGGGACGCC) targeting exon 14 of mouse *Kif6* (ENSMUST00000162854.1) in order to generate a C-terminal truncation allele. The gene specific and universal tracrRNA oligonucleotides (S3 Table) were annealed, filled in with CloneAmp HiFi PCR premix, column purified, and directly used for *in vitro* transcription of single-guide RNAs (sgRNAs) with a T7 Polymerase mix (M0255A NEB). All sgRNA reactions were treated with RNAse free-DNAse. We utilized a ssDNA oligo (S3 Table) to insert a frameshift mutation in all three reading frames, along with 8-cutter restriction sites for genotyping (3-stop donor) [51] (S2 Fig). The *Kif6* ex14 3-stop donor and mKif6-R2-ex14-T7 sgRNA were submitted for pronuclear injection at the University of Texas at Austin Mouse Genetic Engineering Facility (UT-MGEF) using standard protocols (https://www.biomedsupport.utexas.edu/transgenics). We confirmed segregation of the *Kif6*^*p*.*G555fs*^ allele using several methods including increased mobility on a high percentage electrophoresis gel, donor-specific primer PCR, or PmeI (NEB) digestion of the *Kif6* exon14 amplicon (S2 Fig and S3 Table). PCR products in isolated alleles were cloned to pCRII TOPO (ThermoFisher) to identify scarless integration of the 3-stop donor at the *Kif6* locus using gene specific flanking primers (S3 Table).

*Kif6-LacZ*^*tm1b*^ mice were generated by injection of embryonic stem cell clones obtained from the Knockout Mouse Project (KOMP) Repository. Three *Kif6*^*tm1a(KOMP)Mbp*^ embryonic stem (ES) cell clones (KOMP: EPD0736_3_G01; EPD0736_3_H02; and EPD0736_3_A03) all targeting exon 4 of the *Kif6* gene with a promoter-driven targeting cassette for the generation of a 'Knockout-first allele' [52]. Pronuclear injections of all clones were done using standard procedures established by the UT MGEF. After screening for germline transmission, we isolated and confirmed a single heterozygous founder male (*Kif6*^*tm1a(KOMP)Mbp*^) carrier derived from the G01 clone. We confirmed the locus by long-range PCR, several confirmation PCR strategies targeting specific transgene sequences, and Sanger sequencing of the predicted breakpoints (S3 Table). After several backcrosses to the WT C57BL/6J substrain (JAX), we crossed a hemizygous *Kif6*^*tm1a/+*^ mutant male to a homozyogus *CMV-Cre* female (*B6*.*C-Tg(CMV-cre)1Cgn/J*) (JAX, 006054) to convert the *Kif6*^*tm1a*^ allele to a stable LacZ expressing *Kif6*^*tm1b*^ allele (*Kif6*-*LacZ*^*tm1b*^). Mutant F1 offspring from this cross were backcrossed to WT C57BL6/J mice and the F2 progeny were genotyped to confirm the *Kif6*-*LacZ*^*tm1b*^ allele and the presence/absence of the CMV-Cre transgene. A single founder *Kif6-LacZ*^*tm1b*^ with the desired genotype (*Kif6-LacZ*^*tm1b*^ hemizygous, Cre transgene absent) was used to expand a colony for spatial expression analysis.

*Kif6*^*tm1c*^ conditional ready mice were generated by outcross of the *Kif6*^*tm1a(KOMP)Mbp*^ allele described above to a ubiquitously expressed Flippase strain (*129S4/SvJaeSor-Gt(ROSA)26Sor*^*tm1(FLP1)Dym*^*/J*) (JAX, 003946). F1 offspring were genotyped and sequenced at several breakpoints to ensure proper flip recombination and a single F1 founder was used to backcross to C57B6/J for propagation of the *Kif6*^*tm1c*^ strain. Analysis of recombination of the floxed *Kif6*^*tm1c*^ was performed by crossing homozygous *Kif6*^*tm1c/tm1c*^ to a compound heterozygous *CMV-Cre*; *Kif6*^*tm1c/+*^ mouse. Recombination of the exon 4 of Kif6 was confirmed by PCR-gel electrophoresis analysis (S3 Table).

### LacZ staining protocol

Mice were perfused with LacZ fixative and post fixed for 2 hours at RT. Whole brains were then stained in X-gal solution overnight at 37°C followed by post-fixation in 4% PFA overnight at 4°C. The samples were then prepped for cryosectioning in 30% sucrose/OCT and sectioned. Sections were counter stained in Nuclear Fast Red stain (Sigma).

### X-ray analyses of mice

Radiographs of the mouse skeleton were generated using a Kubtec DIGIMUS X-ray system (Kubtec, T0081B) with auto exposure under 25 kV.

### Zebrafish manipulations and transgenesis

All zebrafish studies and procedures were approved by the Animal Studies Committee at the University of Texas at Austin (AUP-2015-00187). Adult zebrafish of the AB were maintained and bred as previously described [53]. Individual fish were used for analysis and compared to siblings and experimental control fish of similar size and age. Independent experiments were repeated using separate clutches of animals. Strains generated for this study: Tg(Foxj1a:GFP)^dp1^ and Tg(foxj1a::Arl13b-GFP)^dp15^. Transgenic lines were generated using a Gateway-compatible middle entry cloning containing mouse *Arl13b* open reading frame[54]was modified to include a C-terminal GFP by megaprimer PCR to generate pME-Arl13b GFP. This clone was recombined with p5E-*foxj1aP* [26], p3E-polyA[55] and pDEST pDestTol2pACryGFP to generate a final transgenesis vector. Embryos were injected at the one-cell stage with 25 pg of assembled transgene and 25 pg Tol2 mRNA. Embryos were sorted at 48 hpf for reporter expression (GFP+ eyes) and were subsequently grown to adulthood. Individuals were bred to TU wild-type zebrafish to generate a stable F1 line, and subsequently bred into a *kif6*^*sko*^ mutant background. pDestTol2pACryGFP was a gift from Joachim Berger & Peter Currie (Addgene plasmid # 64022). Previously published strains: *kif6*^*sko*^ [19].

### Xenopus manipulation and analysis

*Xenopus* embryo manipulations were carried out using standard protocols [56]. Full length of *Xenopus* Kif6 cDNA sequence was provided from Xenbase (www.xenbase.org) and amplified from *Xenopus* cDNA library by PCR and inserted in-frame into pCS10R-eGFP. 5’-capped Kif6-GFP RNA was synthesized using mMESSAGE mMACHINE SP6 transcription kit (Invitrogen Ambion). Synthetic 5’-capped RNAs: Kif6-GFP, membrane RFP and Centrin4-BFP [35] were injected into two ventral blastomeres at the 4-cell stage with ~ 40 pg/RNA/injection. Live images were captured with a Zeiss LSM700 laser scanning confocal microscope using a plan-APOCHROMAT 63X 1.4 NA oil objective lens (Zeiss).

### Mouse and zebrafish perfusions and embedding of brain tissues

Mice were humanely euthanized by extended CO_2_ exposure and transferred to chemical hood where the mouse was perfused with buffered saline followed by 4% PFA. Whole brains were placed in 4% PFA 4 hours at RT, then at 4° C overnight. Zebrafish were euthanized by exposure to lethal, extended dose of Tricane (8%) followed by decapitation. Zebrafish brains were extracted and fixed in 4% PFA at 4° C overnight. For paraffin embedding, the fixed brains were embedded and cut using standard paraffin embedding and sectioning protocols. Paraffin sections were stained with standard hematoxylin-eosin solution.

For frozen sections both mouse or zebrafish brains were fixed as above and then equilibrated to 30% or 35% sucrose, respectively at 4° C overnight. Whole brains were then placed in O.C.T. Compound (Tissue-Tek) and flash in cold ethanol bath. All blocks were stored at -80° Celsius until sectioning on a cryostat (Leica). All sections were dried at RT for ~2hrs. and stored at -80°C until use.

### Immunofluorescence protocol for frozen brain sections

Sectioned tissues were warmed at room temperature for ~1 hour, then washed thrice in 1xPBS + 0.1% Tween (PBST). Antigen retrieval was hot citrate buffer (pH6.8). Blocking was done in 10% Normal goat serum (Sigma) in 1xPBST. Primary antibodies (S100B at 1:1,000, ab52642, Abcam; CD133(Prominin-1), 134A, 1/500; Gamma Tubulin, sc-17787, Santa Cruz (C-11), 1/500; Anti-GFP, SC9996, Santa Cruz, 1:1,000) were diluted in 10% NGSS, 1xPBST and allowed to bind overnight at 4°C in a humidified chamber. Secondary fluorophores (Alexa Fluor 488(A-11034); 568(A10042); and 647(A32728), 1:1,000, ThermoFisher) were diluted in 10% NGS; 1xPBST were allowed to bind at RT for ~1hr. We used Prolong gold with DAPI (Cell Signaling Technologies, 8961) to seal coverslips prior to imaging.

### Iodine-contrast μCT

Zebrafish specimens were fixed overnight in 10% buffered formalin, washed thrice in diH2O and stained ~48 hours in 25% Lugol’s solution/75% distilled water. Specimens were scanned by the High-resolution X-ray CT Facility (http://www.ctlab.geo.utexas.edu/) on an Xradia at 100kV, 10W, 3.5s acquisition time, detector 11.5 mm, source -37 mm, XYZ [816, 10425, –841], camera bin 2, angles ±180, 1261 views, no filter, dithering, no sample drift correction. Reconstructed with center shift 5.5, beam hardening 0.15, theta -7, byte scaling [–150, 2200], binning 1, recon filter smooth (kernel size = 0.5).

### Ex-Vivo ventricle imaging

Brains were isolated from freshly euthanized mice and were dissected in cold DMEM/F12 media. The brain was cut into thin coronal slices, promptly placed onto cover slip with PBS, and imaged using oblique lighting with a Keyence BZ-X800 microscope.

### Statistical analysis and image measures

GraphPad Prism version 7.0c for Mac (GraphPad Software) was used to analyze and plot data. Images for measurement were opened in FIJI (Image J) [57], and measures were taken using the freehand tool to draw outlines on ventricular area or whole brain area. Statistically significant differences between any two groups were examined using a two-tailed Student’s t-test, given equal variance. P values were considered significant at or below 0.05.

## Supporting information

S1 Fig Clustal Alignment of *CPE* (p.V79M) variant. Hs, *Homo sapiens*; Pt, *Pan troglodytes*; Mc, *Macaca mulatta*; Mu, *Mus musculus*; Rn, *Rattus norvegicus*; Bt, *Bos Taurus*; Cl, *Canis lupus familiaris*; Oc, *Oryctolagus cuniculus*; Gg, *Gallus gallus*; Dr, *Danio rerio*; Tn, *Tetraodon nigroviridis*; Xt, *Xenopus (Silurana) tropicalis*; Tc, *Tribolium castaneum*; Ce, *Caenorhabditis elegans*; Sk, *Saccoglossus kowalevskii*.
(TIF)

S2 Fig *Kif6^p.G555fs^* generation and genotyping. (A) Schematic of target cut site and insertion cassette into exon 14 of *Kif6* locus. Insertion cassette contains three stop codons, one in each reading frame, and two 8 basepair restriction enzyme cut sites for easy genotyping. (B-C) Agarose gels of PCR products confirming germline transmission of donor cassette in F_1_ generation from CRISPR injected chimeras. (B) RE digest of PCR product from exon 14 flanking target site, shows cutting (asterisks) in heterozygous F1 mice. Wildtype band (arrow) appears in lane one and all the subsequent lanes. (C) PCR product from donor specific primer and *Kif6* exon 14 reverse primer confirming donor insertion and germline transmission. (D) Table describing CRISPR injected mice, number with detectable indels, total with integration of donor oligo, and total displaying hydrocephaly of chimeric injected CRISPR mice. (E) Germline transmission of donor cassette from chimeric CRISPR F0 mice to F1 generation.
(TIF)

S3 Fig *Cre-CMV; Kif6^fl/fl^* mice display hydrocephaly at P14. (A-B) Dilation of the LV and 3V evident in P14 mice through coronal sections of H&E stained mouse brains from heterozygous and *Cre-CMV; Kif6*^*fl/fl*^ mice. (C) Quantification of LV area over the total brain area shows significant increase in ventricular area in *Cre-CMV;Kif6*^*fl/fl*^ mice.
(TIF)

S4 Fig *LacZ* expression in different ages of *Kif6-LacZ^tm1b^* transgenic mouse brain. (A-A”) Coronal sections of P21 mouse brains showing LacZ staining restricted to the EP cell layer in the 4^th^ ventricle. Zoom in shows LacZ positive cells have cilia projecting into the lumen (arrows). (B-B’) Coronal sections of P21 mouse brains showing LacZ staining of ventral portion of 3^rd^ ventricle. (B’) Some sporadic staining appearing in the nuclei of the hypothalamus (arrows). (C-C’) LacZ staining in the fourth ventricle at P10 showing staining specific to ependymal cell layer.
(TIF)

S5 Fig Immunofluorescence (IF) of Kif6 mutant multiciliated tissues in mouse and zebrafish. (A-B) Immunofluorescence of trachea sections in *Kif6*^*p*.*G555fs/+*^ and *Kif6*^*p*.*G555fs*^ mice showing no apparent cilia defects present in trachea of *Kif6* mutant mice. Acetylated tubulin (green) marking cilia, DAPI-stained nuclei (magenta) (C-D) Representative IF of zebrafish nasal pit cilia shows typical cilia in *kif6* mutant zebrafish to wildtype counterparts. Acetylated tubulin (magenta) marking cilia, gamma-tubulin marking basal bodies (green). Scale bars are 20μM.
(TIF)

S6 Fig SEM of lateral ventricle in *Kif6^p.G555fs^* mutant and control at P28. SEM of *Kif6* wildtype vs. *Kif6*^*p*.*G555fs*^ mutants shows *Kif6* mutants show a complete loss of ependymal cell cilia on the lateral wall by P28. Scale bar 10μM.
(TIF)

S7 Fig Immunofluorescence shows defects in differentiation of EC cilia. (A-B) P14 mouse brains were sectioned and stained in wildtype and *Kif6* mutant tissues to reveal EC cilia never fully differentiate. S100B (magenta) denotes proper specification of ECs in wildtype and mutant tissue, gamma-tubulin (cyan) shows basal bodies docking on the apical surface of ECs in both wildtype and *Kif6* mutants, and finally CD133 (green) shows greatly diminished axonemes in *Kif6*^*p*.*G555fs*^ mutants compared to wildtype controls. (C) Quantitation of maximum intensity projection of fluorescence of the CD133 channel (EC axonemes). Scale bar 20uM.
(TIF)

S8 Fig Kif6 mutant mice have defects in differentiation of EC cilia. (A-D) P14 and P21 *Kif6^p.G555fs^* mutants show reduction in acetylated tubulin in ciliary axonemes. (A and C) P14 and P21 heterozygous littermates show normal EC specification, S100B (magenta), and extension of ciliary axonemes into the lumen of the ventricle, acetylated tubulin (green). (B and D) *Kif6*^p.G555fs^ mutant mice however show a severe reduction in acetylated tubulin in EC ciliary axonemes at both P14 and P21. Scale bar 20uM.
(TIF)

S9 Fig SEM of ventricle in *kif6^sko^* mutant zebrafish display dilation of the ventricular system and loss of ependymal cell cilia. Scanning Electron Microscopy of zebrafish brain shows dilation of rhombencephalic (blue box) and telencephalic (green box) ventricles (red dotted line) indicative of hydrocephaly. Higher magnification images reveal loss of ependymal cell cilia tufts (red arrowheads) in *kif6* zebrafish mutants when compared with heterozygous counterparts (red arrowheads). Scale bars 20μM and 200μM. (CCe: Cerebellum)
(TIF)

S10 Fig Kif6-EGFP can localizes to mitotic spindle poles. A time course of images showing microinjected Kif6-EGFP localizes to microtubule-rich spindle poles and mitotic spindle during cell divisions in the rapidly dividing blastomeres of the early embryo.
(TIF)

S11 Fig Western blot for KIF6 antibodies using mouse lysates from wildtype, *Kif6^p.G555fs^*, and *Cre-CMV;Kif6^fl/fl^*. Representative testing of two different KIF6 antibodies in mouse ventricular lysates failed to show a banding at reported size, or banding patterns at a size different to that of what is reported. Vinculin antibody used as a loading control.
(TIF)

S12 Fig X-rays of *Kif6^p.G555fs^* mutant mice. Representative X-rays of wildtype and *Kif6*^*p*.*G555fs*^ and wildtype mutant mice shows no scoliosis at P28. *Kif6*^*p*.*G555fs*^ mice do however display skull expansion caused by progressive hydrocephalus (Animal #1 and #2).
(TIF)

S1 Table Eighty-three homozygous variants from WES. (DOCX)

S2 Table Sixty-three homozygous regions from homozygosity mapping. (DOCX)

S3 Table Mouse specific oligos and primers. (DOCX)

S4 Table Numerical data and summary statistics. (XLSX)

S1 Movie Ex vivo oblique bright field imaging of *Kif6^p.G555fs/+^* heterozygous mouse ventricle. (MP4)

S2 Movie Ex vivo oblique bright field imaging of *Kif6^p.G555fs^* mutant mouse ventricle. (MP4)

S3 Movie Representative WT_Danio_Iodine-contrasted microCT transverse slices. (AVI)

S4 Movie Representative *kif6^sko^*_Danio_Iodine-contrasted microCT transverse slices. (AVI)
