# Supplementary figures and images for: Mutations in Kinesin family member 6 reveal specific role in ependymal cell ciliogenesis and human neurological development
Source: PLoS Genet. 2018 Nov 26;14(11):e1007817. doi: 10.1371/journal.pgen.1007817 (PMC6307780; doi:10.1371/journal.pgen.1007817)

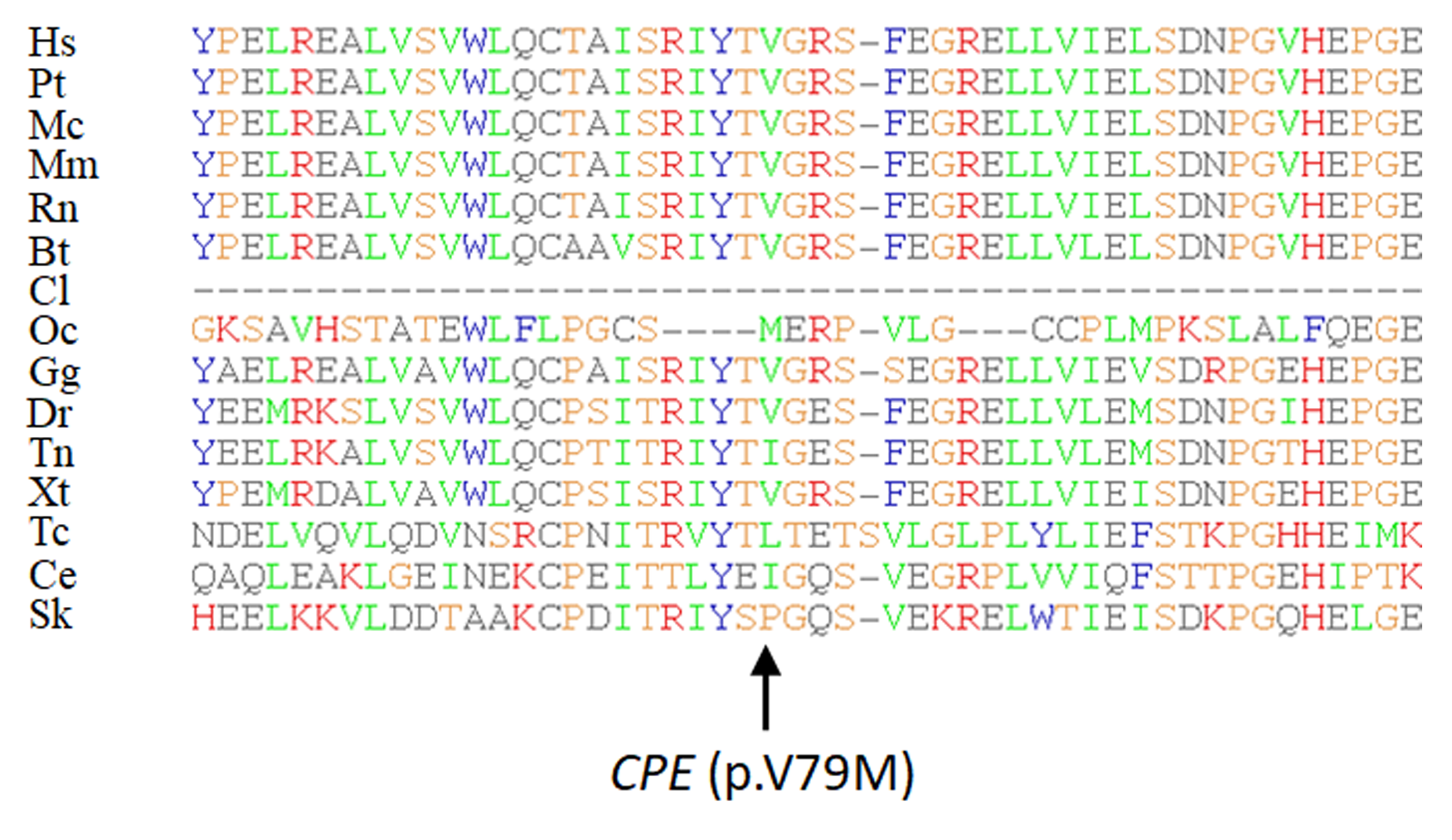

Supplement: S1 Fig — Hs, Homo sapiens; Pt, Pan troglodytes; Mc, Macaca mulatta; Mu, Mus musculus; Rn, Rattus norvegicus; Bt, Bos Taurus; Cl, Canis lupus familiaris; Oc, Oryctolagus cuniculus; Gg, Gallus gallus; Dr, Danio rerio; Tn, Tetraodon nigroviridis; Xt, Xenopus (Silurana) tropicalis; Tc, Tribolium castaneum; Ce, Caenorhabditis elegans; Sk, Saccoglossus kowalevskii. (TIF) [file pgen.1007817.s001.tif]

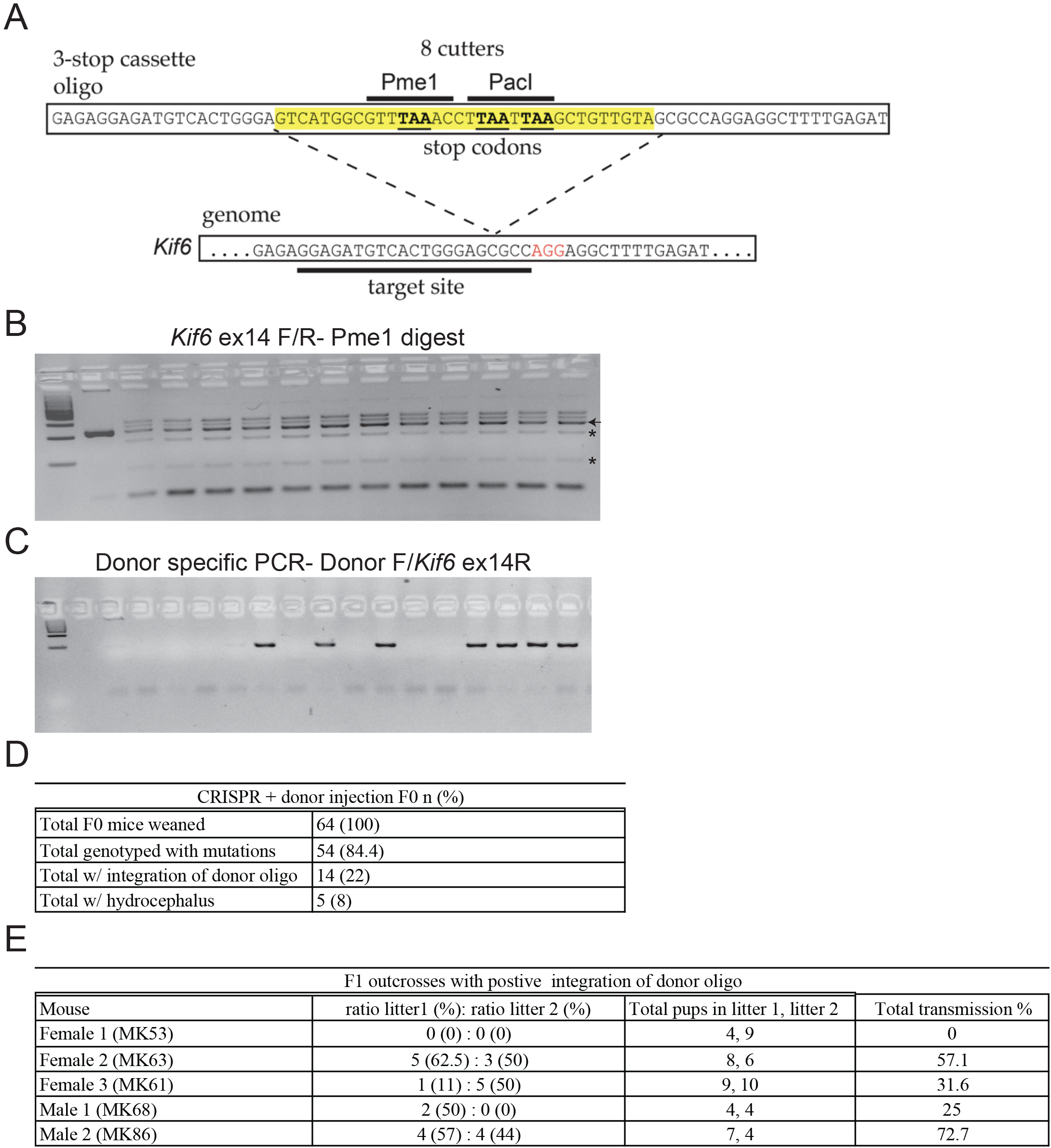

Supplement: S2 Fig — (A) Schematic of target cut site and insertion cassette into exon 14 of Kif6 locus. Insertion cassette contains three stop codons, one in each reading frame, and two 8 basepair restriction enzyme cut sites for easy genotyping. (B-C) Agarose gels of PCR products confirming germline transmission of donor cassette in F1 generation from CRISPR injected chimeras. (B) RE digest of PCR product from exon 14 flanking target site, shows cutting (asterisks) in heterozygous F1 mice. Wildtype band (arrow) appears in lane one and all the subsequent lanes. (C) PCR product from donor specific primer and Kif6 exon 14 reverse primer confirming donor insertion and germline transmission. (D) Table describing CRISPR injected mice, number with detectable indels, total with integration of donor oligo, and total displaying hydrocephaly of chimeric injected CRISPR mice. (E) Germline transmission of donor cassette from chimeric CRISPR F0 mice to F1 generation. (TIF) [file pgen.1007817.s002.tif]

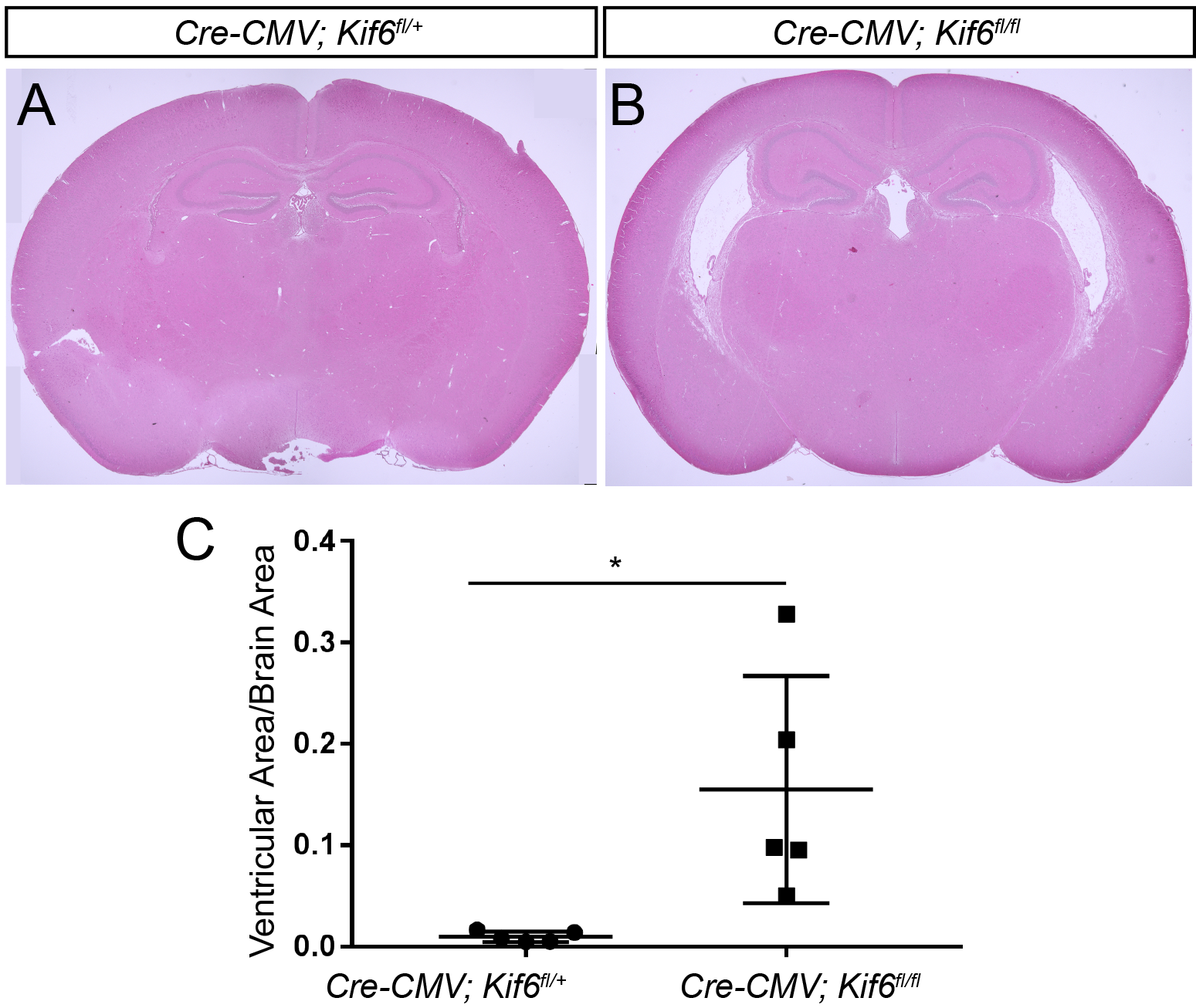

Supplement: S3 Fig — (A-B) Dilation of the LV and 3V evident in P14 mice through coronal sections of H&E stained mouse brains from heterozygous and Cre-CMV; Kif6fl/fl mice. (C) Quantification of LV area over the total brain area shows significant increase in ventricular area in Cre-CMV;Kif6fl/fl mice. (TIF) [file pgen.1007817.s003.tif]

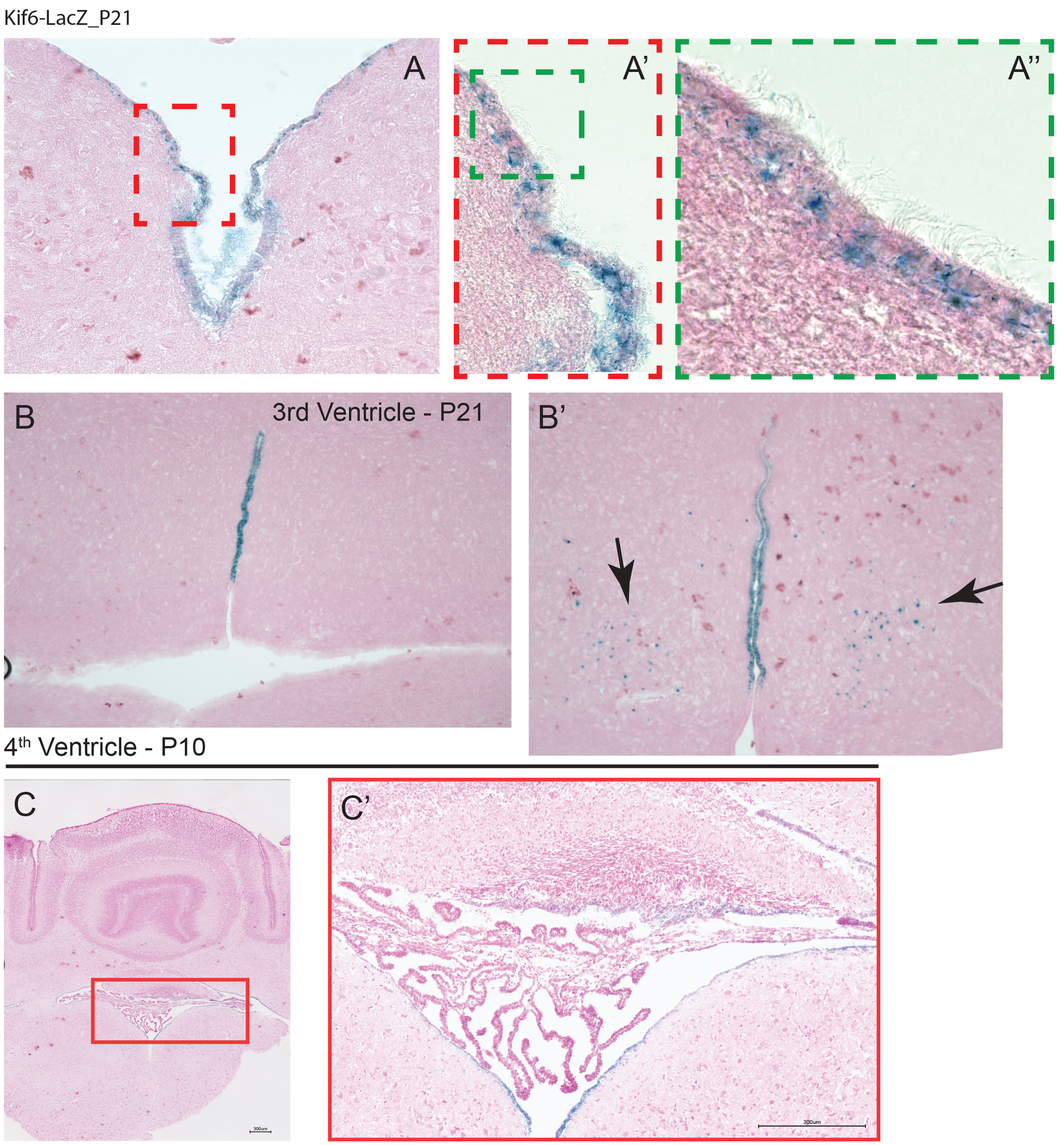

Supplement: S4 Fig — (A-A”) Coronal sections of P21 mouse brains showing LacZ staining restricted to the EP cell layer in the 4th ventricle. Zoom in shows LacZ positive cells have cilia projecting into the lumen (arrows). (B-B’) Coronal sections of P21 mouse brains showing LacZ staining of ventral portion of 3rd ventricle. (B’) Some sporadic staining appearing in the nuclei of the hypothalamus (arrows). (C-C’) LacZ staining in the fourth ventricle at P10 showing staining specific to ependymal cell layer. (TIF) [file pgen.1007817.s004.tif]

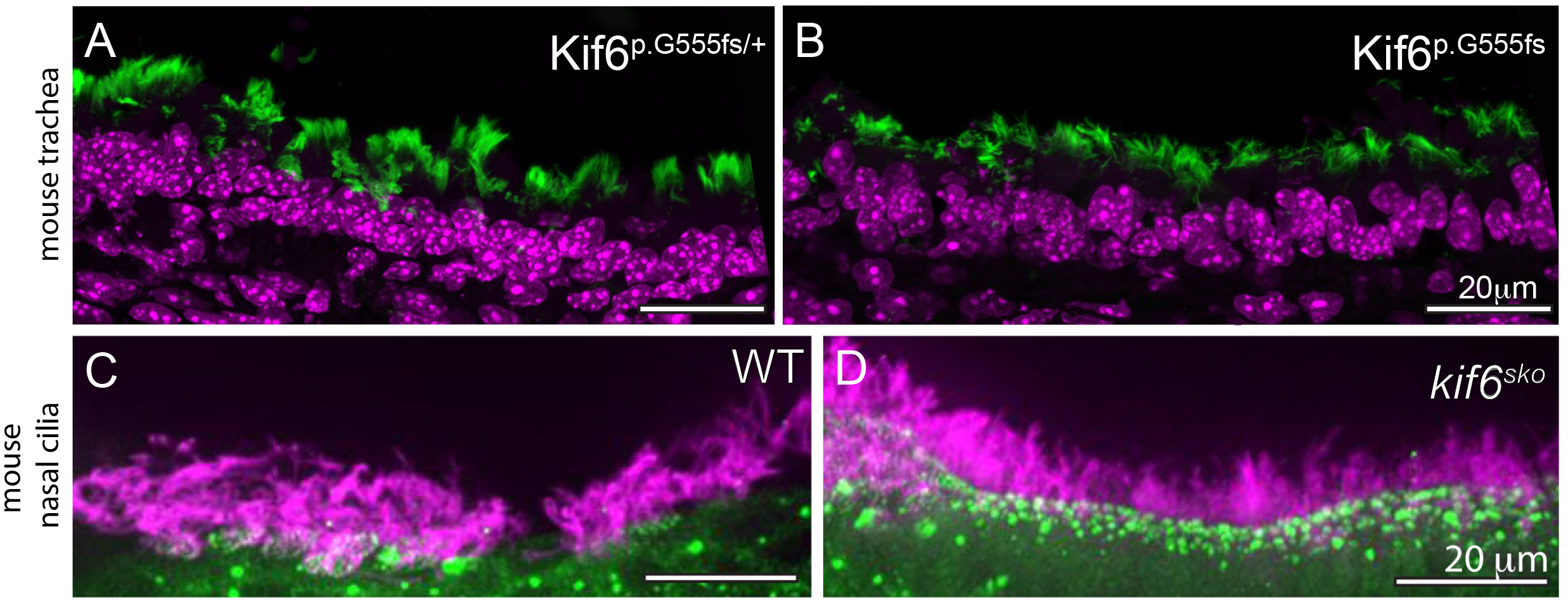

Supplement: S5 Fig — (A-B) Immunofluorescence of trachea sections in Kif6p.G555fs/+ and Kif6p.G555fs mice showing no apparent cilia defects present in trachea of Kif6 mutant mice. Acetylated tubulin (green) marking cilia, DAPI-stained nuclei (magenta) (C-D) Representative IF of zebrafish nasal pit cilia shows typical cilia in kif6 mutant zebrafish to wildtype counterparts. Acetylated tubulin (magenta) marking cilia, gamma-tubulin marking basal bodies (green). Scale bars are 20μM. (TIF) [file pgen.1007817.s005.tif]

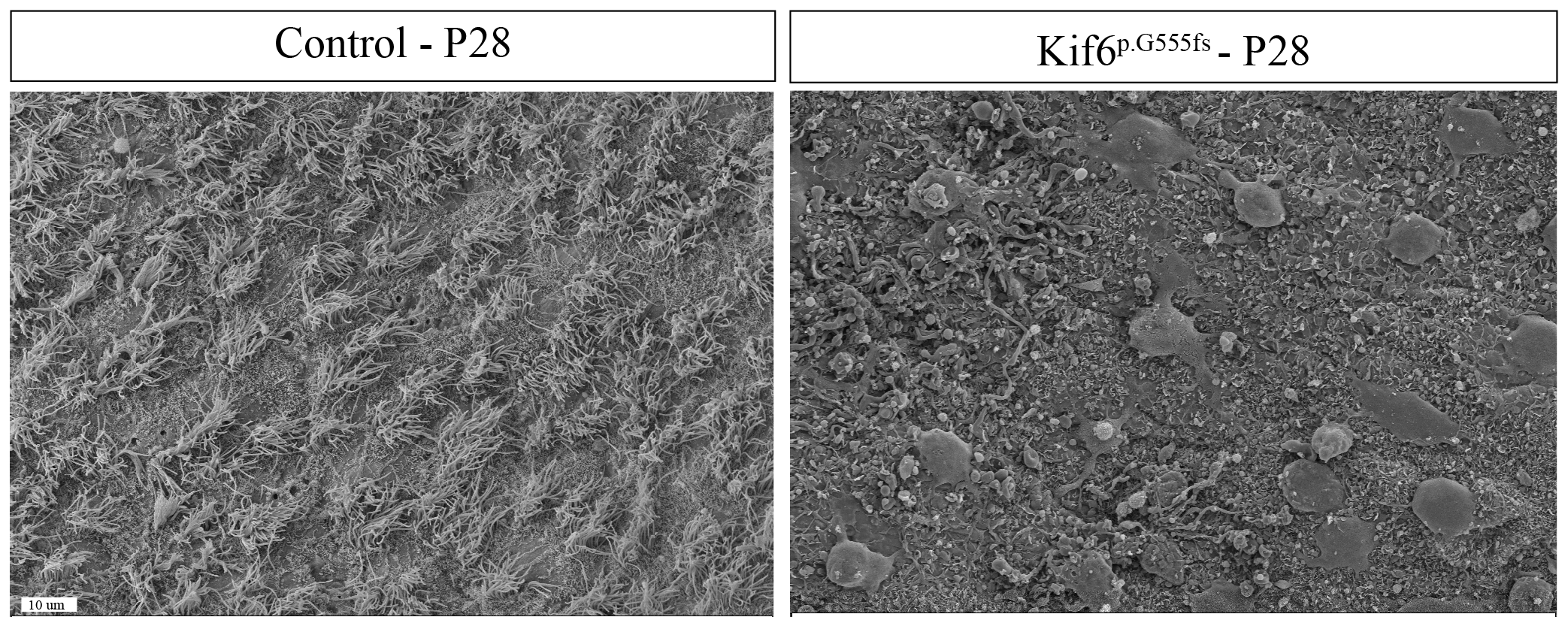

Supplement: S6 Fig — SEM of Kif6 wildtype vs. Kif6p.G555fs mutants shows Kif6 mutants show a complete loss of ependymal cell cilia on the lateral wall by P28. Scale bar 10μM. (TIF) [file pgen.1007817.s006.tif]

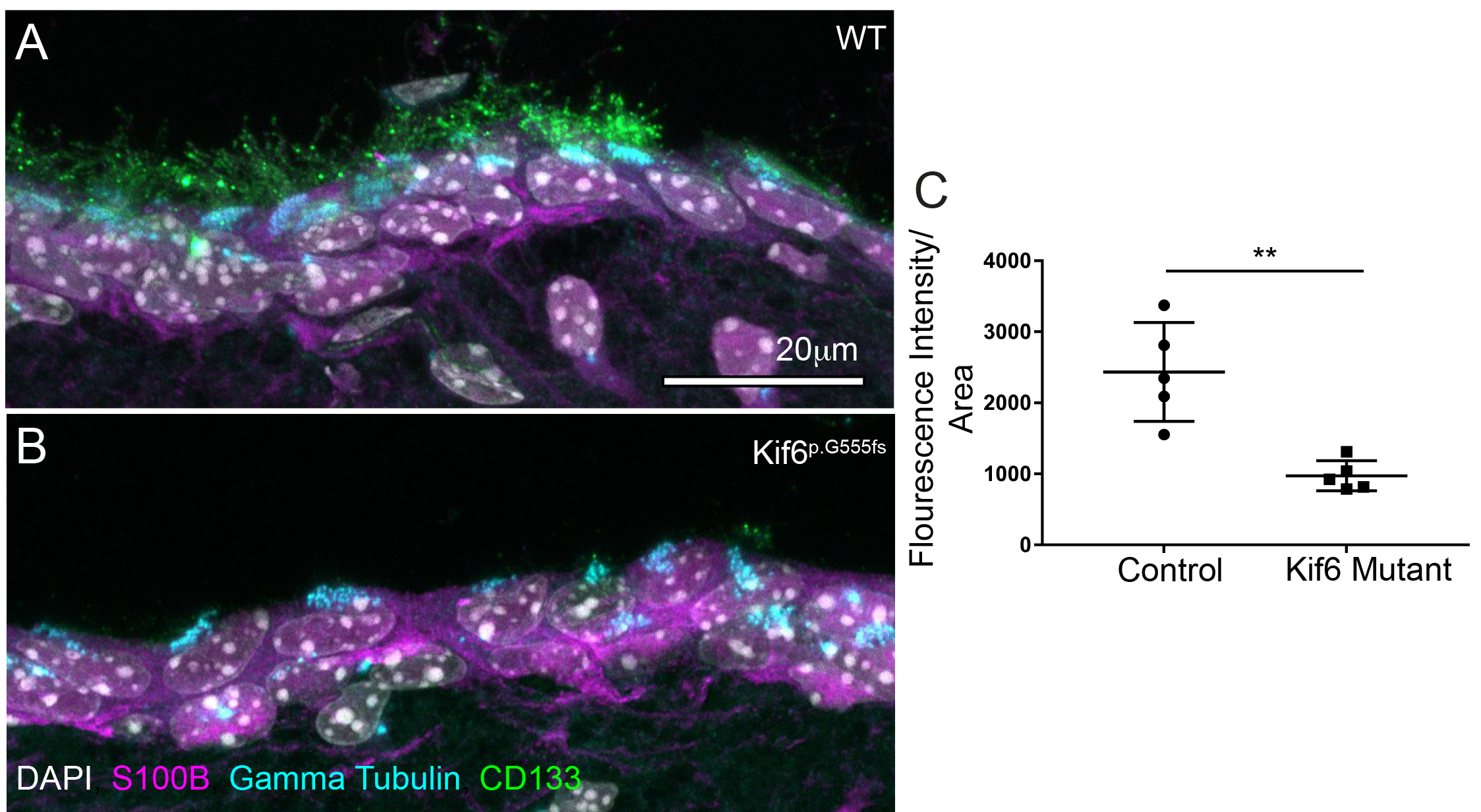

Supplement: S7 Fig — (A-B) P14 mouse brains were sectioned and stained in wildtype and Kif6 mutant tissues to reveal EC cilia never fully differentiate. S100B (magenta) denotes proper specification of ECs in wildtype and mutant tissue, gamma-tubulin (cyan) shows basal bodies docking on the apical surface of ECs in both wildtype and Kif6 mutants, and finally CD133 (green) shows greatly diminished axonemes in Kif6p.G555fs mutants compared to wildtype controls. (C) Quantitation of maximum intensity projection of fluorescence of the CD133 channel (EC axonemes). Scale bar 20uM. (TIF) [file pgen.1007817.s007.tif]

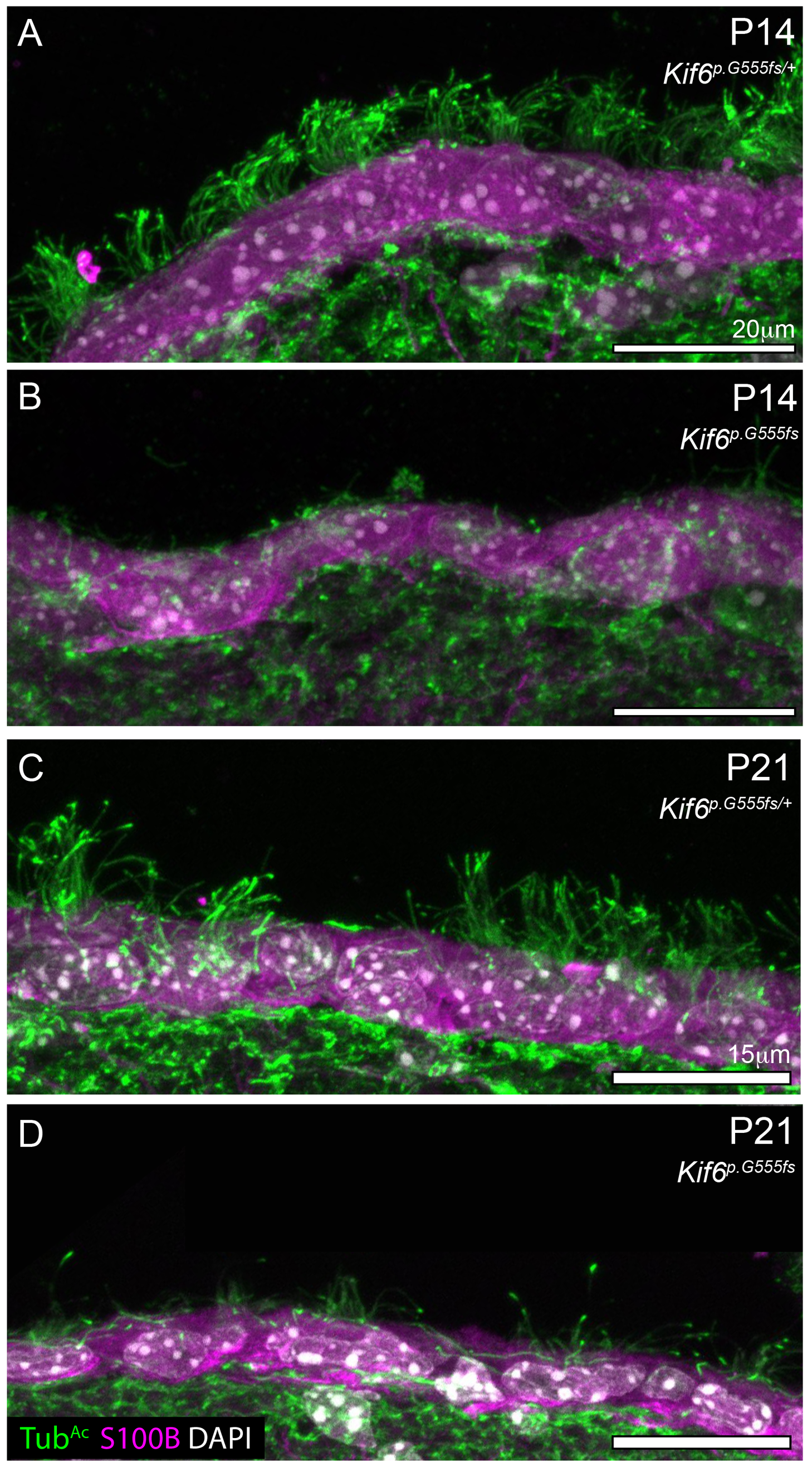

Supplement: S8 Fig — (A-D) P14 and P21 Kif6p.G555fs mutants show reduction in acetylated tubulin in ciliary axonemes. (A and C) P14 and P21 heterozygous littermates show normal EC specification, S100B (magenta), and extension of ciliary axonemes into the lumen of the ventricle, acetylated tubulin (green). (B and D) Kif6p.G555fs mutant mice however show a severe reduction in acetylated tubulin in EC ciliary axonemes at both P14 and P21. Scale bar 20uM. (TIF) [file pgen.1007817.s008.tif]

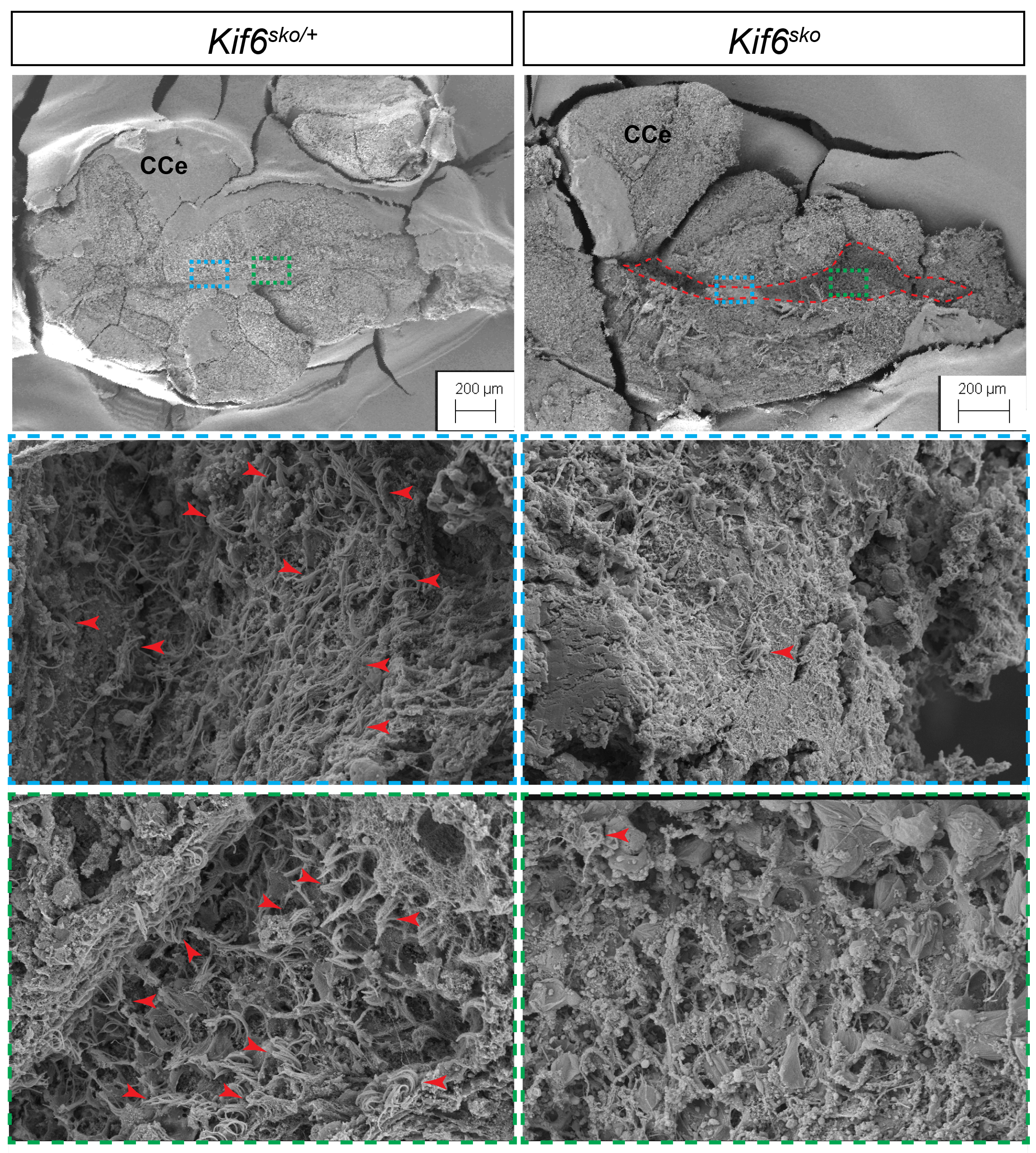

Supplement: S9 Fig — Scanning Electron Microscopy of zebrafish brain shows dilation of rhombencephalic (blue box) and telencephalic (green box) ventricles (red dotted line) indicative of hydrocephaly. Higher magnification images reveal loss of ependymal cell cilia tufts (red arrowheads) in kif6 zebrafish mutants when compared with heterozygous counterparts (red arrowheads). Scale bars 20μM and 200μM. (CCe: Cerebellum) (TIF) [file pgen.1007817.s009.tif]

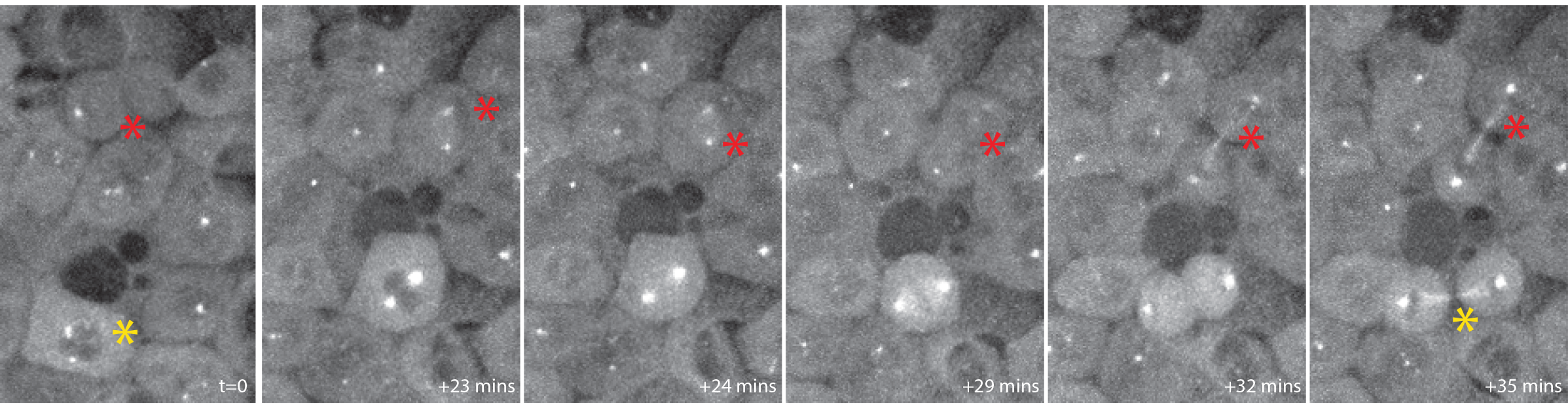

Supplement: S10 Fig — A time course of images showing microinjected Kif6-EGFP localizes to microtubule-rich spindle poles and mitotic spindle during cell divisions in the rapidly dividing blastomeres of the early embryo. (TIF) [file pgen.1007817.s010.tif]

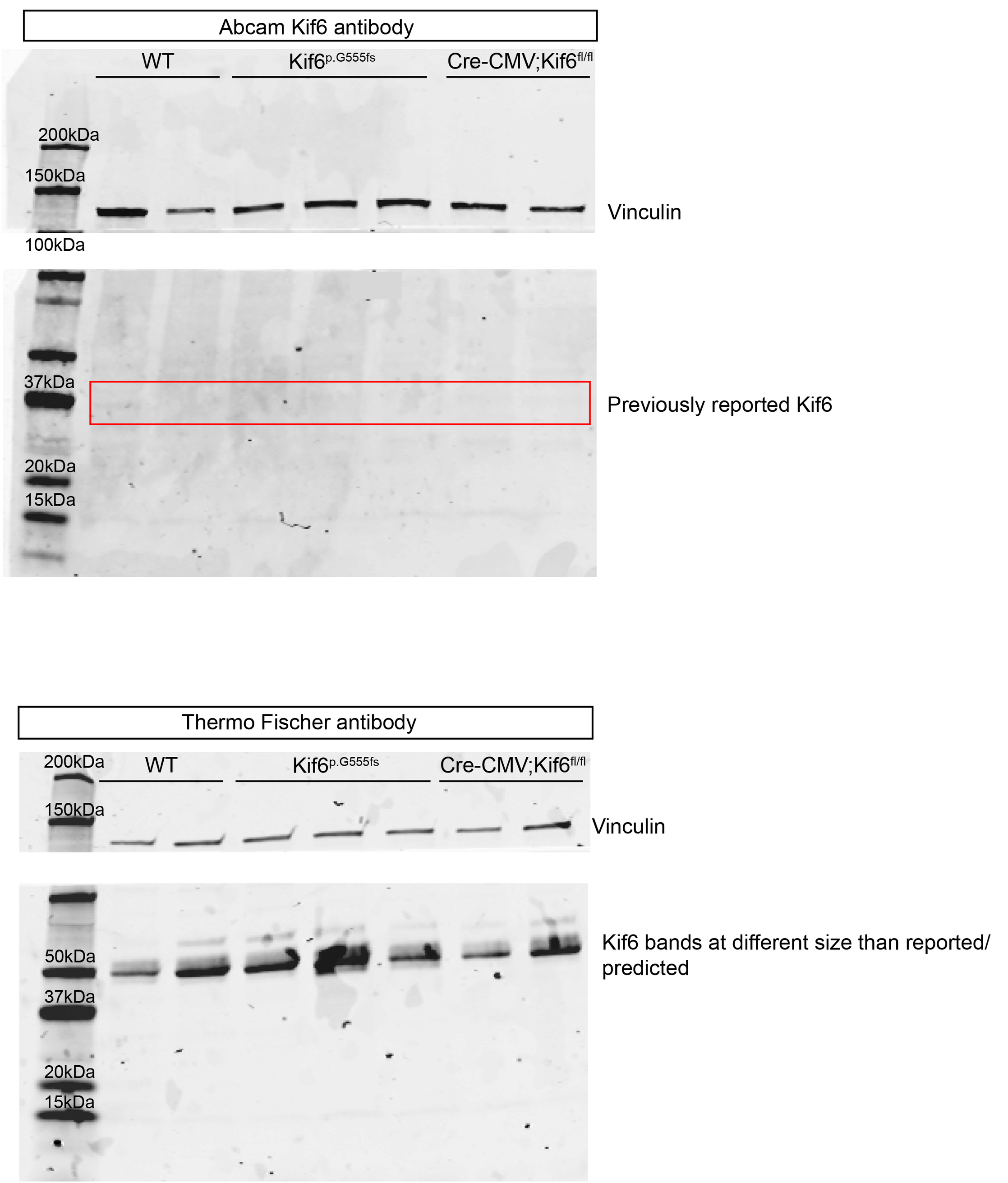

Supplement: S11 Fig — Representative testing of two different KIF6 antibodies in mouse ventricular lysates failed to show a banding at reported size, or banding patterns at a size different to that of what is reported. Vinculin antibody used as a loading control. (TIF) [file pgen.1007817.s011.tif]

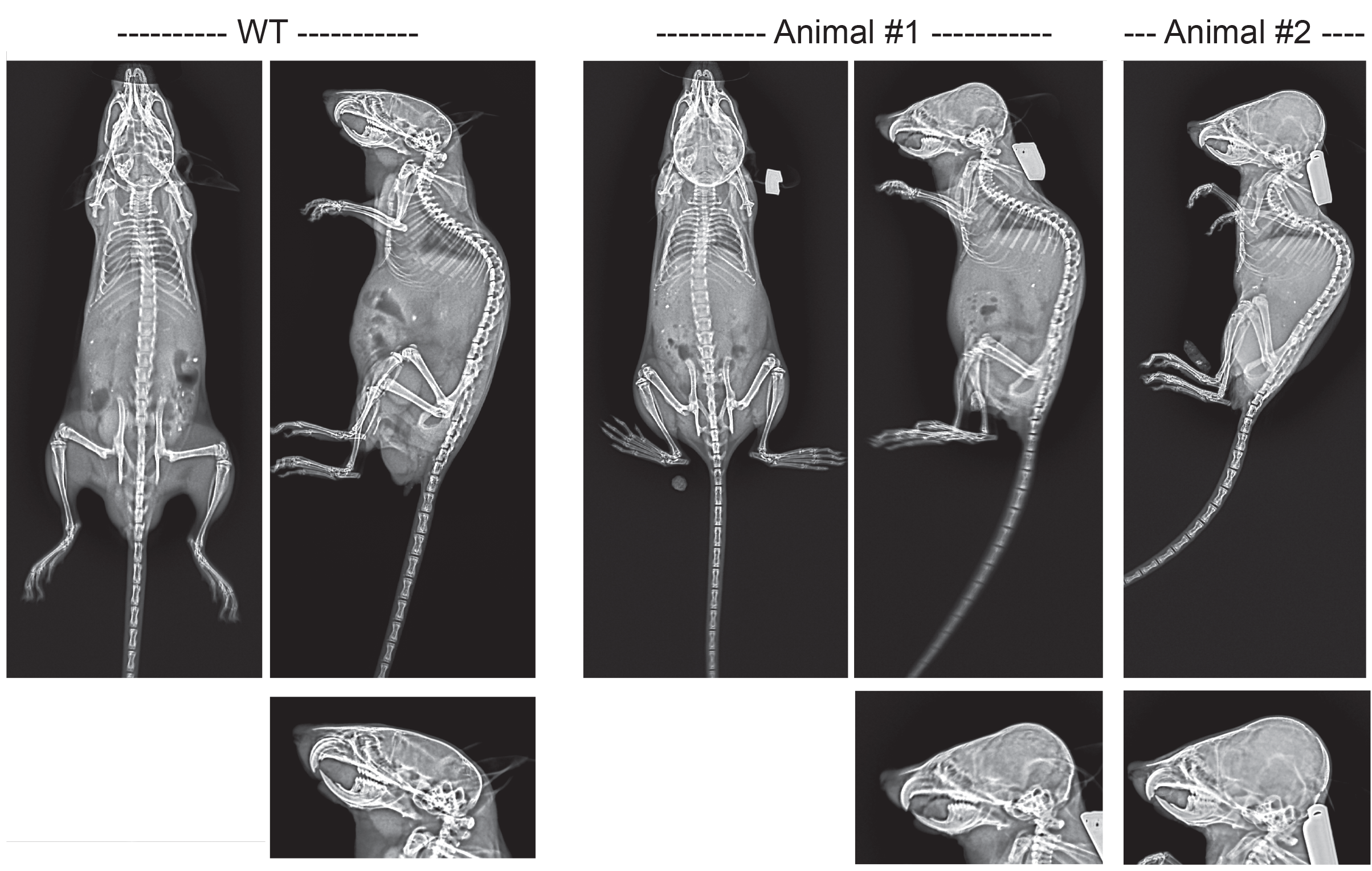

Supplement: S12 Fig — Representative X-rays of wildtype and Kif6p.G555fs and wildtype mutant mice shows no scoliosis at P28. Kif6p.G555fs mice do however display skull expansion caused by progressive hydrocephalus (Animal #1 and #2). (TIF) [file pgen.1007817.s012.tif]
