# Supplementary material for: Mutations in Kinesin family member 6 reveal specific role in ependymal cell ciliogenesis and human neurological development
Source: PLoS Genet. 2018 Nov 26;14(11):e1007817. doi: 10.1371/journal.pgen.1007817 (PMC6307780; doi:10.1371/journal.pgen.1007817)
Supplement: S1 Table — (DOCX) [file pgen.1007817.s013.docx]

**Supplementary Table SI. Eighty three homozygous variants from WES**

| Chromosome | Position_start | Position_end | Gene | Change |
| --- | --- | --- | --- | --- |
| 1 | 26608891 | 26608896 | *UBXN11* | nonframeshift_deletion |
| 1 | 54605319 | 54605319 | *CDCP2* | frameshift_insertion |
| 1 | 152681694 | 152681694 | *LCE4A* | nonframeshift_insertion |
| 1 | 156565050 | 156565050 | *GPATCH4* | frameshift_insertion |
| 1 | 160650923 | 160650923 | *CD48* | frameshift_deletion |
| 2 | 96148317 | 96148317 | *TRIM43B* | nonsynonymous_SNV |
| 2 | 130902532 | 130902532 | *CCDC74B* | nonsynonymous_SNV |
| 2 | 240981597 | 240981597 | *PRR21* | nonsynonymous_SNV |
| 2 | 240981627 | 240981627 | *PRR21* | nonsynonymous_SNV |
| 2 | 240981655 | 240981655 | *PRR21* | nonsynonymous_SNV |
| 2 | 241621800 | 241621800 | *AQP12B* | frameshift_deletion |
| 3 | 40503552 | 40503552 | *RPL14* | nonframeshift_insertion |
| 3 | 53324829 | 53324834 | *DCP1A* | nonframeshift_deletion |
| 3 | 56650056 | 56650056 | *CCDC66* | nonframeshift_insertion |
| 3 | 75786586 | 75786586 | *ZNF717* | nonsynonymous_SNV |
| 3 | 75786662 | 75786662 | *ZNF717* | nonsynonymous_SNV |
| 3 | 75786672 | 75786672 | *ZNF717* | nonsynonymous_SNV |
| 3 | 75786684 | 75786684 | *ZNF717* | nonsynonymous_SNV |
| 3 | 75786764 | 75786764 | *ZNF717* | frameshift_deletion |
| 3 | 75790810 | 75790810 | *ZNF717* | frameshift_insertion |
| 3 | 195505930 | 195505930 | *MUC4* | nonsynonymous_SNV |
| 3 | 195506099 | 195506099 | *MUC4* | nonsynonymous_SNV |
| 3 | 195506147 | 195506147 | *MUC4* | nonsynonymous_SNV |
| 3 | 195506156 | 195506156 | *MUC4* | nonsynonymous_SNV |
| 3 | 195506267 | 195506267 | *MUC4* | nonsynonymous_SNV |
| 3 | 195506507 | 195506507 | *MUC4* | nonsynonymous_SNV |
| 3 | 195506530 | 195506530 | *MUC4* | nonsynonymous_SNV |
| 3 | 195506704 | 195506704 | *MUC4* | nonsynonymous_SNV |
| 3 | 195507226 | 195507226 | *MUC4* | nonsynonymous_SNV |
| 3 | 195510310 | 195510310 | *MUC4* | nonsynonymous_SNV |
| 3 | 195510613 | 195510613 | *MUC4* | nonsynonymous_SNV |
| 3 | 195512042 | 195512042 | *MUC4* | nonsynonymous_SNV |
| 3 | 195514558 | 195514558 | *MUC4* | nonsynonymous_SNV |
| 3 | 195514733 | 195514733 | *MUC4* | nonsynonymous_SNV |
| 3 | 195514768 | 195514768 | *MUC4* | nonsynonymous_SNV |
| 4 | 166300608 | 166300608 | *CPE* | nonsynonymous_SNV |
| 5 | 139931628 | 139931628 | *SRA1* | frameshift_insertion |
| 5 | 140307142 | 140307142 | *PCDHAC1* | nonsynonymous_SNV |
| 5 | 140725635 | 140725635 | *PCDHGA3* | nonsynonymous_SNV |
| 5 | 149512332 | 149512332 | *PDGFRB* | nonsynonymous_SNV |
| 6 | 16327953 | 16327955 | *ATXN1* | nonframeshift_deletion |
| 6 | 39513453 | 39513453 | *KIF6* | frameshift_deletion |
| 6 | 41895234 | 41895234 | *BYSL* | nonsynonymous_SNV |
| 6 | 43970526 | 43970531 | *C6orf223* | nonframeshift_deletion |
| 7 | 1586661 | 1586661 | *TMEM184A* | nonframeshift_insertion |
| 7 | 15725824 | 15725826 | *MEOX2* | nonframeshift_deletion |
| 7 | 44040763 | 44040763 | *SPDYE1* | nonsynonymous_SNV |
| 7 | 44120345 | 44120345 | *POLM* | nonsynonymous_SNV |
| 7 | 64167644 | 64167644 | *ZNF107* | nonsynonymous_SNV |
| 7 | 64169019 | 64169019 | *ZNF107* | frameshift_insertion |
| 7 | 100639147 | 100639147 | *MUC12* | nonsynonymous_SNV |
| 7 | 100639153 | 100639153 | *MUC12* | nonsynonymous_SNV |
| 7 | 100646440 | 100646440 | *MUC12* | nonsynonymous_SNV |
| 7 | 100647338 | 100647338 | *MUC12* | nonsynonymous_SNV |
| 7 | 100647339 | 100647339 | *MUC12* | nonsynonymous_SNV |
| 7 | 100647376 | 100647376 | *MUC12* | nonsynonymous_SNV |
| 7 | 143270465 | 143270465 | *CTAGE15P* | nonsynonymous_SNV |
| 8 | 7629232 | 7629232 | *FAM90A10* | nonsynonymous_SNV |
| 8 | 144511981 | 144511983 | *MAFA* | nonframeshift_deletion |
| 9 | 79318378 | 79318392 | *PRUNE2* | nonframeshift_deletion |
| 9 | 90534190 | 90534202 | *FAM75C1* | frameshift_substitution |
| 9 | 90746922 | 90746922 | *FAM75C2* | nonsynonymous_SNV |
| 9 | 97080947 | 97080949 | *FAM22F* | nonframeshift_deletion |
| 9 | 107367666 | 107367667 | *OR13C2* | frameshift_deletion |
| 10 | 27342292 | 27342294 | *ANKRD26* | nonframeshift_deletion |
| 10 | 50535007 | 50535007 | *C10orf71* | frameshift_insertion |
| 10 | 119302954 | 119302956 | *EMX2* | nonframeshift_deletion |
| 10 | 135438687 | 135438687 | *FRG2B* | nonsynonymous_SNV |
| 10 | 135438929 | 135438929 | *FRG2B* | nonsynonymous_SNV |
| 10 | 135438943 | 135438943 | *FRG2B* | nonsynonymous_SNV |
| 10 | 135438961 | 135438961 | *FRG2B* | nonsynonymous_SNV |
| 11 | 65636053 | 65636053 | *EFEMP2* | nonsynonymous_SNV |
| 13 | 72440664 | 72440664 | *DACH1* | nonsynonymous_SNV |
| 15 | 34825154 | 34825154 | *GOLGA8B* | nonsynonymous_SNV |
| 15 | 82637448 | 82637448 | *GOLGA6L10* | nonsynonymous_SNV |
| 19 | 1556482 | 1556482 | *MEX3D* | nonsynonymous_SNV |
| 19 | 13318707 | 13318712 | *CACNA1A* | nonframeshift_deletion |
| 19 | 20807178 | 20807178 | *ZNF626* | frameshift_insertion |
| 19 | 22156850 | 22156850 | *ZNF208* | nonsynonymous_SNV |
| 19 | 46627337 | 46627337 | *IGFL3* | nonsynonymous_SNV |
| 20 | 126314 | 126315 | *DEFB126* | frameshift_deletion |
| 20 | 238439 | 238444 | *DEFB132* | nonframeshift_deletion |
| 20 | 32664865 | 32664865 | *RALY* | nonframeshift_insertion |
