# Supplementary material for: Mutations in Kinesin family member 6 reveal specific role in ependymal cell ciliogenesis and human neurological development
Source: PLoS Genet. 2018 Nov 26;14(11):e1007817. doi: 10.1371/journal.pgen.1007817 (PMC6307780; doi:10.1371/journal.pgen.1007817)
Supplement: S2 Table — (DOCX) [file pgen.1007817.s014.docx]

**Supplementary Table SII. Sixty three homozygous regions from homozygosity mapping**

| Chromosome | SNP1 | SNP2 | Position 1 | Position 2 | Size (Kb) |
| --- | --- | --- | --- | --- | --- |
| 1 | kgp9226796 | kgp15281431 | 42,272,542 | 43,304,041 | 1031.499 |
| 1 | rs12043872 | kgp2176506 | 50,644,891 | 52,222,874 | 1577.983 |
| 1 | kgp22807508 | kgp15446070 | 175,901,363 | 177,085,495 | 1184.132 |
| 2 | rs13383059 | kgp1338077 | 60,542,657 | 61,840,186 | 1297.529 |
| 2 | kgp22835335 | rs1345516 | 63,283,437 | 64,703,286 | 1419.849 |
| 2 | kgp10464820 | kgp11645441 | 72,356,303 | 73,389,904 | 1033.601 |
| 2 | kgp22748117 | rs11683207 | 95,350,864 | 98,333,290 | 2982.426 |
| 2 | kgp11241972 | kgp14721953 | 110,452,502 | 111,457,965 | 1005.463 |
| 2 | kgp14255701 | kgp4421106 | 155,497,134 | 156,765,691 | 1268.557 |
| 3 | rs2242150 | kgp920744 | 48,505,964 | 50,176,739 | 1670.775 |
| 3 | rs1528197 | kgp5972268 | 62,404,892 | 63,669,341 | 1264.449 |
| 3 | kgp7194326 | rs17024881 | 84,835,064 | 86,118,949 | 1283.885 |
| 3 | rs6804377 | kgp10736192 | 89,271,087 | 90,501,225 | 1230.138 |
| 3 | kgp17732198 | kgp3463695 | 186,780,268 | 195,465,310 | 8685.042 |
| 3 | rs3747673 | kgp22812098 | 195,611,844 | 197,891,568 | 2279.724 |
| 4 | rs922333 | kgp20990514 | 64,121,298 | 65,135,489 | 1014.191 |
| 4 | kgp12219459 | kgp21246176 | 155,003,651 | 182,666,898 | 27663.247 |
| 5 | rs11948368 | kgp6573465 | 4,225,004 | 10,668,729 | 6443.725 |
| 5 | kgp3769852 | rs13184580 | 42,912,540 | 43,918,326 | 1005.786 |
| 5 | kgp3714973 | kgp12345716 | 132,628,429 | 141,015,519 | 8387.09 |
| 5 | rs41098 | kgp22527688 | 141,020,100 | 149,585,074 | 8564.974 |
| 5 | kgp22340708 | kgp10344299 | 149,587,580 | 172,127,143 | 22539.563 |
| 5 | kgp22126080 | kgp3444559 | 172,127,834 | 173,730,182 | 1602.348 |
| 6 | rs12663002 | rs3094575 | 28,441,634 | 29,515,802 | 1074.168 |
| 6 | kgp9471913 | kgp17000708 | 34,187,366 | 43,010,582 | 8823.216 |
| 6 | rs9342711 | kgp8490913 | 69,197,250 | 70,393,636 | 1196.386 |
| 6 | rs6926330 | kgp17199538 | 91,038,726 | 108,320,258 | 17281.532 |
| 6 | kgp6823635 | kgp10978549 | 126,186,643 | 127,216,752 | 1030.109 |
| 7 | rs17714729 | kgp13497896 | 40,925,373 | 44,100,951 | 3175.578 |
| 7 | kgp13393169 | kgp1758811 | 44,282,204 | 58,042,660 | 13760.456 |
| 7 | kgp13613108 | kgp7238344 | 61,055,273 | 63,041,496 | 1986.223 |
| 7 | kgp22797960 | kgp1628657 | 63,234,502 | 64,474,466 | 1239.964 |
| 7 | kgp8836768 | kgp6578278 | 64,476,704 | 66,158,076 | 1681.372 |
| 7 | kgp11959682 | kgp731589 | 66,835,845 | 73,110,455 | 6274.61 |
| 7 | kgp13574770 | kgp13241424 | 73,254,871 | 75,614,264 | 2359.393 |
| 7 | rs782487 | rs41542 | 76,161,400 | 93,722,036 | 17560.636 |
| 8 | rs35292150 | rs10097659 | 7,154,036 | 8,241,316 | 1087.28 |
| 8 | kgp20383958 | kgp20415047 | 47,840,086 | 50,247,347 | 2407.261 |
| 8 | kgp4568187 | kgp4989542 | 50,287,180 | 51,332,763 | 1045.583 |
| 8 | kgp11163141 | kgp8120576 | 83,652,903 | 84,739,570 | 1086.667 |
| 8 | kgp1752758 | rs6468704 | 99,405,919 | 100,994,389 | 1588.47 |
| 8 | rs1318739 | kgp3429874 | 104,122,872 | 105,175,235 | 1052.363 |
| 8 | kgp20054401 | kgp10669054 | 114,857,226 | 115,996,248 | 1139.022 |
| 9 | kgp3876298 | kgp18506353 | 66,771,643 | 71,032,042 | 4260.399 |
| 10 | kgp22751182 | kgp22827934 | 73,974,125 | 75,401,246 | 1427.121 |
| 10 | kgp28675 | kgp1752039 | 128,613,842 | 133,791,976 | 5178.134 |
| 11 | rs7129994 | kgp12754305 | 84,380,034 | 85,620,335 | 1240.301 |
| 12 | kgp1178574 | rs7311759 | 111,816,925 | 113,261,665 | 1444.74 |
| 13 | kgp7808335 | rs8002509 | 36,452,369 | 47,822,976 | 11370.607 |
| 13 | rs9568798 | kgp16817285 | 53,614,554 | 54,687,807 | 1073.253 |
| 13 | kgp9865216 | kgp16686988 | 61,860,814 | 62,932,723 | 1071.909 |
| 13 | kgp7743232 | rs9523513 | 65,234,035 | 92,781,385 | 27547.35 |
| 13 | kgp1128060 | rs16953570 | 96,133,164 | 97,438,801 | 1305.637 |
| 15 | rs4508402 | rs4779824 | 30,361,587 | 31,404,294 | 1042.707 |
| 15 | kgp19953534 | kgp19917981 | 64,125,067 | 65,168,281 | 1043.214 |
| 16 | kgp6578578 | kgp22822345 | 31,888,867 | 33,579,417 | 1690.55 |
| 16 | kgp10923077 | kgp16403964 | 46,920,923 | 48,045,778 | 1124.855 |
| 17 | kgp22779423 | kgp14115230 | 57,513,947 | 59,233,842 | 1719.895 |
| 18 | kgp9512908 | kgp6363611 | 51,184,594 | 52,469,636 | 1285.042 |
| 19 | rs1144539 | kgp7929656 | 37,331,614 | 38,475,123 | 1143.509 |
| 20 | kgp19251773 | rs6061136 | 29,423,716 | 30,644,465 | 1220.749 |
| 20 | kgp19300395 | rs2425193 | 32,830,486 | 34,848,116 | 2017.63 |
| 21 | kgp6761551 | kgp7702343 | 41,544,215 | 44,477,135 | 2932.92 |
