# Supplementary material for: Mutations in Kinesin family member 6 reveal specific role in ependymal cell ciliogenesis and human neurological development
Source: PLoS Genet. 2018 Nov 26;14(11):e1007817. doi: 10.1371/journal.pgen.1007817 (PMC6307780; doi:10.1371/journal.pgen.1007817)
Supplement: S3 Table — (DOCX) [file pgen.1007817.s015.docx]

| ***Kif6^tm1a^*^(KOMP)Wtsi^**  **Cassette primers** |  |
| --- | --- |
| CSD-lacF | GCTACCATTACCAGTTGGTCTGGTGTC |
| CSD-neoF | GGGATCTCATGCTGGAGTTCTTCG |
| CSD-loxF | GAGATGGCGCAACGCAATTAATG |
| ***Kif6 ^tm1a^*^(KOMP)Wtsi^**  **Gene Specific Primers** |  |
| CSD-Kif6-R | GGTTAGGAGGAAGAGAAGGGCATCC |
| CSD-Kif6-ttR | ACAGATGCTGGAGATCACACTCTCG |
| CSD-Kif6-F | ACTCTCTTCAAAGCCCACATCATGC |
| **Mouse CRISPR oligos/genotyping primers** |  |
| mKif6-R2-ex14-T7 | TAATACGACTCACTATAGGAGATGTCACTGGGACGCCGTTTTAGAGCTAGAAATAGC |
| Universal T7 tracer | AAAAGCACCGACTCGGTGCCACTTTTTCAAGTTGATAACGGACTAGCCTTATTTTAACTTGCTATTTCTAGCTCTAAAAC |
| Ms_*Kif6*crispr_r2_donor oligo | GAGAGGAGATGTCACTGGGAGTCATGGCGTTTAAACCTTAATTAAGCTGTTGTAGCGCCAGGAGGCTTTTGAGAT |
| Mus_*Kif6*_ex14F | TCCCAAAATGATGTGACTGAAG |
| Mus_*Kif6*_ex14R | AGTCTCTGGACTGGCTTACCTG |
| *Kif6p.G555fs* 3stopDonor_FWD | CATGGCGTTTAAACCTTAATTAAGCTG |
| *Kif6p.G555fs* 3stopDonor_REV | CAGCTTAATTAAGGTTTAAACGCCATG |
| **Mouse Kif6 qPCR primer sets** |  |
| Ms_*Kif6*qPCR_exon8_FWD1 | TCGGAAAAACACCGTACACA |
| Ms_*Kif6*qPCR_exon10_REV1 | CTTTTGCAAGCGAACAATCA |
| Ms_*Kif6*qPCR_exon7_FWD2 | TTCAACCCGGTCACACTGTA |
| Ms_*Kif6*qPCR_exon8_REV2 | TACGGTGTTTTTCCGAAAGG |
| Ms_*Kif6*qPCR_exon6_FWD3 | TGGAGGACCCTGATCAGAAC |
| Ms_*Kif6*qPCR_exon7_REV3 | TACAGTGTGACCGGGTTGAA |

Konjikusic et al., 2018

Table SIII
